# SIRT7 remodels the cytoskeleton *via* RAC1 to enhance host resistance to *Mycobacterium tuberculosis*

**DOI:** 10.1128/mbio.00756-24

**Published:** 2024-09-17

**Authors:** Fuxiang Li, Ximeng Zhang, Jinjin Xu, Yue Zhang, Guo Li, Xirui Yang, Guofang Deng, Youchao Dai, Baohua Liu, Christian Kosan, Xinchun Chen, Yi Cai

**Affiliations:** 1 Guangdong Provincial Key Laboratory of Regional Immunity and Diseases, Department of Pathogen Biology, Shenzhen University Medical School, Shenzhen, China; 2 Department of Biochemistry, Center for Molecular Biomedicine (CMB), Friedrich Schiller University Jena, Jena, Germany; 3 School of Pharmaceutical Sciences, Shenzhen University Medical School, Shenzhen, China; 4 Department of Dermatology, Xiangya Hospital, Central South University, Changsha, China; 5 Dornsife College of Letters, Arts and Sciences, University of Southern California, Los Angeles, California, USA; 6 Guangdong Key Lab for Diagnosis & Treatment of Emerging Infectious Diseases, Shenzhen Third People’s Hospital, Shenzhen, China; 7 Guangzhou Eighth People’s Hospital, Guangzhou Medical University, Guangzhou, China; 8 Shenzhen Key Laboratory for Systemic Aging and Intervention (SAI), National Engineering Research Center for Biotechnology (Shenzhen), International Cancer Center, Shenzhen University, Shenzhen, China; The Hebrew University of Jerusalem, Rehobot, Israel

**Keywords:** tuberculosis, macrophages, actin cytoskeleton, phagocytosis, SIRT7, RAC1

## Abstract

Phagocytosis of *Mycobacterium tuberculosis* (*Mtb*) followed by its integration into the matured lysosome is critical in the host defense against tuberculosis. How *Mtb* escapes this immune attack remains elusive. In this study, we unveiled a novel regulatory mechanism by which SIRT7 regulates cytoskeletal remodeling by modulating RAC1 activation. We discovered that SIRT7 expression was significantly reduced in CD14^+^ monocytes of TB patients. *Mtb* infection diminished SIRT7 expression by macrophages at both the mRNA and protein levels. SIRT7 deficiency impaired actin cytoskeleton-dependent macrophage phagocytosis, LC3II expression, and bactericidal activity. In a murine tuberculosis model, SIRT7 deficiency detrimentally impacted host resistance to *Mtb*, while *Sirt7* overexpression significantly increased the host defense against *Mtb*, as determined by bacterial burden and inflammatory–histopathological damage in the lung. Mechanistically, we demonstrated that SIRT7 limits *Mtb* infection by directly interacting with and activating RAC1, through which cytoskeletal remodeling is modulated. Therefore, we concluded that SIRT7, in its role regulating cytoskeletal remodeling through RAC1, is critical for host responses during *Mtb* infection and proposes a potential target for tuberculosis treatment.

**IMPORTANCE:**

Tuberculosis (TB), caused by *Mycobacterium tuberculosis* (*Mtb*), remains a significant global health issue. Critical to macrophages’ defense against *Mtb* is phagocytosis, governed by the actin cytoskeleton. Previous research has revealed that *Mtb* manipulates and disrupts the host’s actin network, though the specific mechanisms have been elusive. Our study identifies a pivotal role for SIRT7 in this context: *Mtb* infection leads to reduced SIRT7 expression, which, in turn, diminishes RAC1 activation and consequently impairs actin-dependent phagocytosis. The significance of our research is that SIRT7 directly engages with and activates Rac Family Small GTPase 1 (RAC1), thus promoting effective phagocytosis and the elimination of *Mtb*. This insight into the dynamic between host and pathogen in TB not only broadens our understanding but also opens new avenues for therapeutic development.

## INTRODUCTION

*Mycobacterium tuberculosis* (*Mtb*), the primary etiological agent of tuberculosis (TB), perpetuates as a predominant global infectious disease that accounted for 1.3 million deaths worldwide in 2022, a year that saw over 10 million new cases of active TB (1). The pathology of TB is directly linked to the tight interplay between the host immune system and the *Mtb* infection (2). To develop new therapeutics for TB, a better understanding of the complex relationship between *Mtb* and its human host is ultimately needed.

Host resistance against *Mtb* depends on the activation of both adaptive and innate immune mechanisms. Macrophages are primary innate immune cells that mediate resistance to TB (3). Phagocytosis by macrophages is a hallmark of the anti-bacterial host defense, yet it is also a process that results in *Mtb* infection (3, 4). After recognizing the pathogen-associated molecular patterns of *Mtb* through pattern-recognition receptors, macrophages initiate signaling cascades that drive the actin-based cytoskeletal remodeling for microbial uptake (5). This process facilitates the formation of phagosomes, which are marked by molecules such as LC3 to promote their transport and fusion with lysosomes (6). The formation of a phagocytic cup, as well as the transport and maturation of phagosomes, necessitates dynamic and extensive assembly and disassembly of actin filaments (7–11). The ability of *Mtb* to adapt and thrive intracellularly relies on the variety of strategies it has evolved to alter host innate immune mechanisms. In particular, interference with phagosome biogenesis has been highlighted as a significant aspect of *Mtb* persistence and replication within macrophages (12). Pathogenic mycobacteria have been shown to interfere with and disrupt the actin filament network of host cells, thereby delaying the acquisition of phagosomal maturation markers and altering the endocytic transport system through actin filament dysfunction, circumventing *Mtb* eradication by macrophages (7, 13–15). Consistent with this, actin filament network disruption by cytochalasin D hinders the movement of early endosomes and probably disrupts fusion events between early endosomes and phagosomes, promoting *Mtb* replication within macrophages (7, 14, 16). Numerous studies have reported the diminished phagocytic capabilities of macrophages derived from elderly populations (17–19), diabetic patients (20, 21), and individuals living with HIV (22, 23). Their compromised macrophage function has been intrinsically linked to the increased susceptibility of these populations to TB (22, 24, 25). In addition, in TB patients, impaired phagocytosis caused by TB drugs (rifampicin and rifabutin) impacts host defenses and thereby influences therapy outcomes (26). Actin filament-network-mediated phagocytosis plays a crucial role in effective TB control. However, the precise host mechanisms regulating cytoskeletal remodeling in *Mtb* infections remain to be elucidated.

In this study, we discovered that Sirtuin 7 (SIRT7), a member of the Sirtuin family of NAD^+^-dependent protein deacetylases (27), participates in cytoskeletal remodeling during *Mtb* infection. We found that SIRT7 expression is significantly decreased during *Mtb* infection in mRNA expression and protein levels. The *SIRT7* deficiency impairs macrophage LC3-associated phagocytosis and bactericidal activity by disrupting actin cytoskeleton dynamics *via* Rac family small GTPase 1 (RAC1) signaling. Importantly, we found that SIRT7 deficiency compromised the host’s response to *Mtb* by increasing the bacterial burden and inflammation in the lungs. By contrast, *SIRT7* overexpression impeded bacterial growth. Therefore, our findings demonstrate a novel role for SIRT7 as a regulator of host–pathogen interactions *via* its modulation and control of the actin cytoskeleton.

## RESULTS

### *Mtb* infection downregulates SIRT7 expression

**Fig 1:**
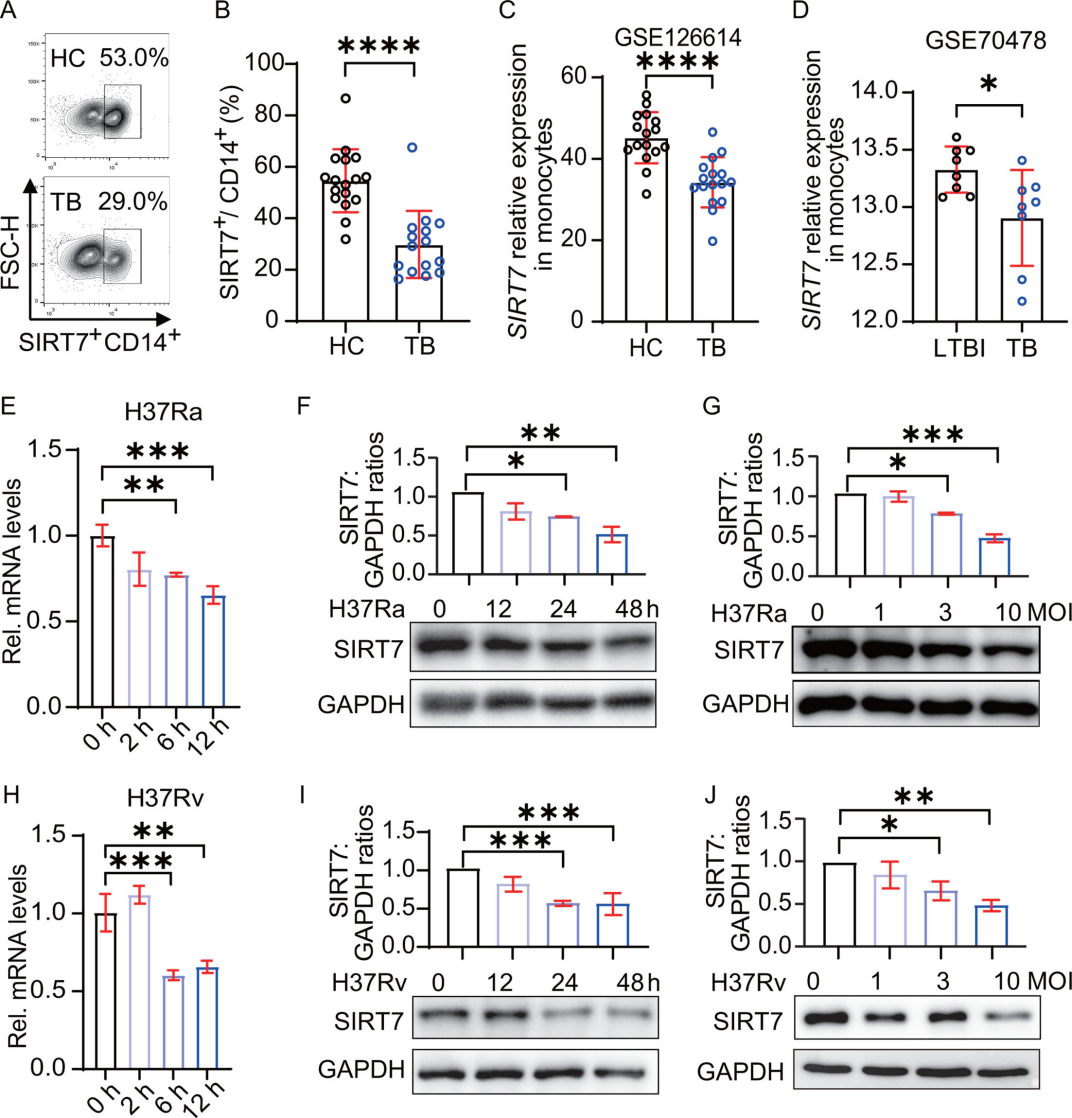
*Mtb* infection downregulates SIRT7 expression. (**A**) Flow cytometry histograms representing SIRT7 expression in monocytes (CD14^+^CD3^-^) from peripheral blood, stained with anti-CD3, anti-CD14, and anti-SIRT7 antibodies followed by a fluorescein isothiocyanate (FITC)-conjugated secondary antibody. SIRT7-positive cells are highlighted within the designated gate. (**B**) Quantification of SIRT7 expression in monocytes from healthy controls (HCs) (*n* = 17) and tuberculosis (TB) patients (*n* = 15). (**C**) Transcripts Per Kilobase Million (TPM)-normalized expression values of *SIRT7* in monocytes from HC (*n* = 16) and TB (*n* = 16) subjects based on the GSE126614 data set. (**D**) Expression values of the *SIRT7* in monocytes from LTBI (*n* = 8) and TB (*n* = 8) subjects based on the GSE70476 data set. (**E and H**) RT-qPCR analysis of *SIRT7* expression at 0, 2, 6, and 12 hours post-infection in THP1-derived macrophages infected with H37Ra and BMDMs infected with H37Rv (MOI = 10). (F, G, I, and J) Immunoblot analysis of SIRT7 expression was conducted at 0, 12, 24, and 48 hours post-infection in THP1-derived macrophages infected with H37Ra, and at 24 hours post-infection in BMDMs infected with H37Rv, across indicated MOI levels (0, 1, 3, or 10). Data are presented as means ± SEM, *P < 0.05, **P < 0.01, ***P < 0.001, ****P < 0.0001, as determined by one-way ANOVA with Tukey’s multiple comparisons test (E through J) or Student’s two-tailed unpaired t-test (B through D). Experiments were performed in triplicate.

We first analyzed the SIRT7 expression profiles of healthy controls (HC) and tuberculosis patients (TB). Utilizing flow cytometry, we determined the SIRT7 levels in various cell types. The results revealed that the percentage of SIRT7 in CD14^+^ monocytes was lower in TB than in HC (Fig. 1A and B; Fig. S1A). However, no significant difference in CD3^+^ T-cell SIRT7 expression was observed between TB and HC (Fig. S1B). This finding was further substantiated by another published RNA-sequencing (RNA-seq) data set, which recorded a notable decline in *SIRT7* expression in monocytes from TB patients relative to those from HC (Fig. 1C), while SIRT7 expression in whole blood presented no discernible variance between the two groups (Fig. S1C). Moreover, previously published microarray data reveal that *SIRT7* expression was diminished in monocytes from TB patients relative to those from individuals with latent TB infection (LTBI) (Fig. 1D), but no concomitant decrease in *SIRT7* expression was identified in the whole blood of TB patients when contrasted with that of the LTBI group (Fig. S1D). Consistent with this, *Mtb* infection significantly reduced SIRT7 expression at both the mRNA and protein levels in a time- and dose-dependent manner in THP1-derived macrophages (Fig. 1E through G; Fig. S1E). A similar trend was observed in bone-marrow-derived macrophages (BMDMs) infected with the H37Rv strain (Fig. 1H through J; Fig. S1F). Specifically, *Mtb* at 3 and 10 multiplicity of infection (MOI) significantly suppressed SIRT7 levels, whereas 1 MOI had no significant effect. Time course analysis revealed no significant changes at 12 hours, whereas marked reductions were evident by 24 hours, intensifying by 48 hours. Cumulatively, our findings underscore the influence of *Mtb* infection on SIRT7 expression and suggest there is a link between diminished SIRT7 expression and TB pathogenesis.

### SIRT7 deficiency increases host susceptibility to *Mtb* infection

**Fig 2:**
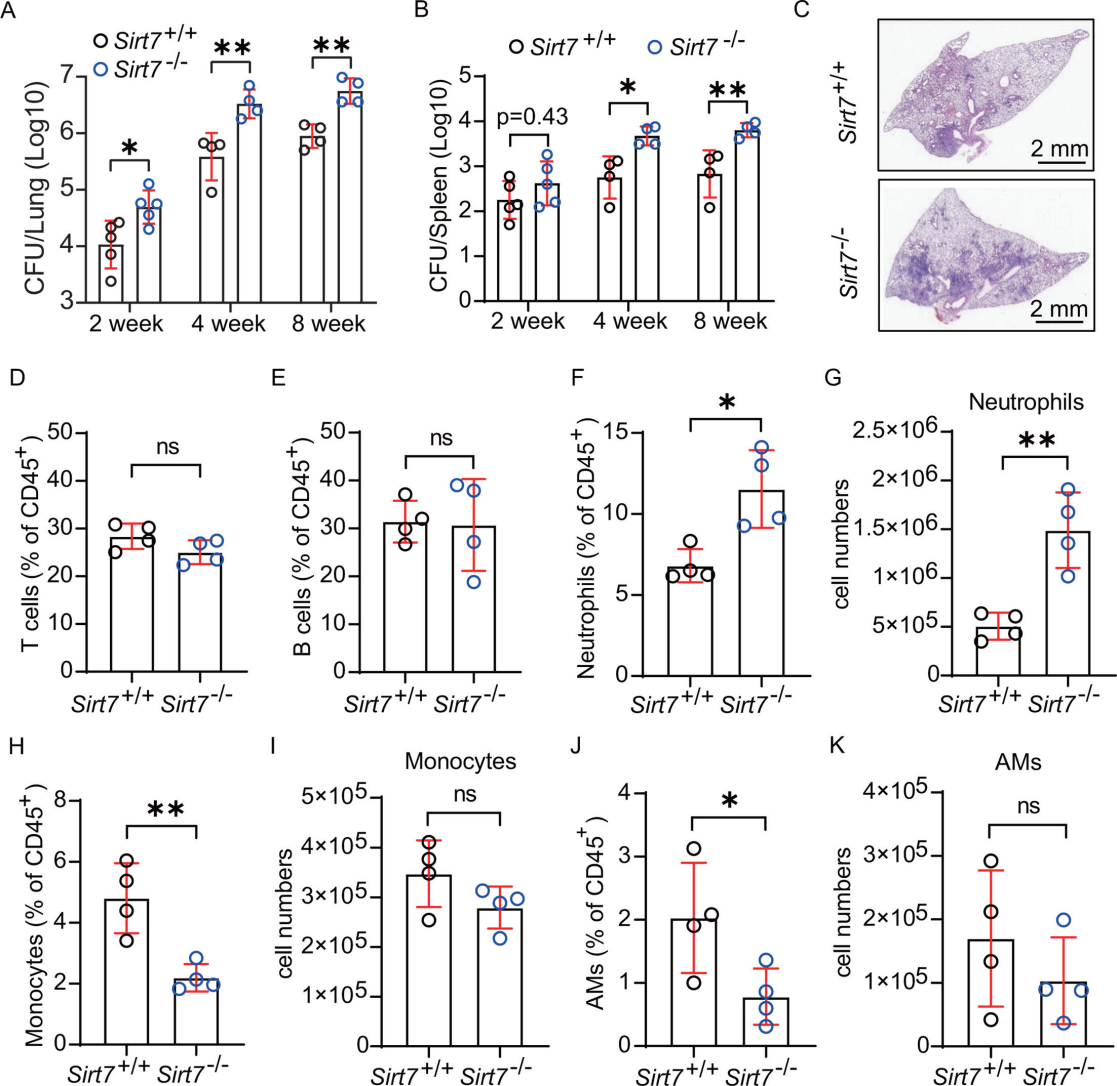
SIRT7 deficiency increases host susceptibility to *Mtb* infection. Mice were infected with *Mtb* strain H37Rv and sacrificed at 14, 28, and 56 days post-infection for the assessment of bacterial load in the lung and spleen, immune cell populations, and histopathology of the lung. (**A and B**) Bacterial burden in the lungs (**A**) and spleens (**B**) of H37Rv-infected *Sirt7*^−/−^ (blue circles) and *Sirt7*^+/+^ (black circles) mice, as assessed *via* CFU counts from tissue homogenates. (**C**) Histopathological analysis of lung sections from *Sirt7*^−/−^ and *Sirt7*^+/+^ mice infected with *Mtb* for 28 days, stained with hematoxylin and eosin. Images were captured using a NanoZoomer digital pathology system (Hamamatsu Photonics). (D through K) Single-cell suspensions prepared from collected lungs were counted and stained for flow cytometry analysis. Percentages (normalized to total CD45+ cells) and absolute numbers of neutrophils (CD45^+^CD19^-^CD11B^+^Ly6G^+^Ly6C^+^), monocytes (CD45^+^CD19^-^CD11B^+^Ly6G^-^Ly6C^+^), alveolar macrophages (AMs, CD45^+^Ly6G^-^siglecf^+^), T cells (CD45^+^CD19^-^CD3^+^), and B cells (CD45^+^CD19^+^) in the lungs from H37Rv-infected *Sirt7*^−/−^ and *Sirt7*^+/+^ mice, 28 days post-infection, were determined. Data are presented as means ± SEM; ns, not significant; *P < 0.05, **P < 0.01, as determined by one-way ANOVA with Tukey’s multiple comparisons test (**A and B**) or Student’s two-tailed unpaired t-test (D through K). Each experiment was independently replicated two to three times.

Our preceding results suggested there is an association between SIRT7 and TB pathogenesis. So, to investigate the role of *Sirt7* in host resistance to *Mtb* infection, we utilized *Sirt7* knockout mice (*Sirt7*^−/−^). We found that, compared to wild-type (*Sirt7*^+/+^) mice, *Sirt7*^−/−^ mice showed increased bacterial burden in lungs and spleens at both 4 and 8 weeks post-infection with H37Rv (Fig. 2A and B). Histological analysis revealed the augmentation of inflammation within the lungs of H37Rv-infected *Sirt7*^−/−^ mice compared to those of *Sirt7*^+/+^ mice (Fig. 2C), indicating that SIRT7 deficiency heightened host susceptibility to TB. No difference was found in CD3 or CD19 cell populations in the lungs of *Sirt7*^−/−^ and *Sirt7*^+/+^ mice (Fig. 2D and E; Fig. S2B and C). However, SIRT7 deficiency resulted in a significant reduction in the proportions of monocytes and macrophages in the lungs, but an increase in both the proportion and absolute cell counts of neutrophils, of H37Rv-infected mice (Fig. 2F through K). To investigate the cause of increased neutrophils in Mtb-infected *Sirt7*^−/−^ mice, we measured CXCL1 and CXCL2 levels in lung homogenate supernatants. CXCL1 levels were significantly higher in Mtb-infected *Sirt7*^−/−^ mice compared to Mtb-infected *Sirt7*^+/+^ mice. While CXCL2 levels did not show a significant difference, they exhibited a similar trend (Fig. S2D and E). In summary, these results suggest that the presence of SIRT7 enhances the ability of mice to inhibit the growth of *Mtb* and ameliorate the pulmonary inflammation triggered by TB infection. Collectively, these results support the hypothesis that SIRT7 has a protective role in defenses against *Mtb* infection.

### SIRT7 deficiency impairs macrophage phagocytosis and bactericidal capacity

**Fig 3:**
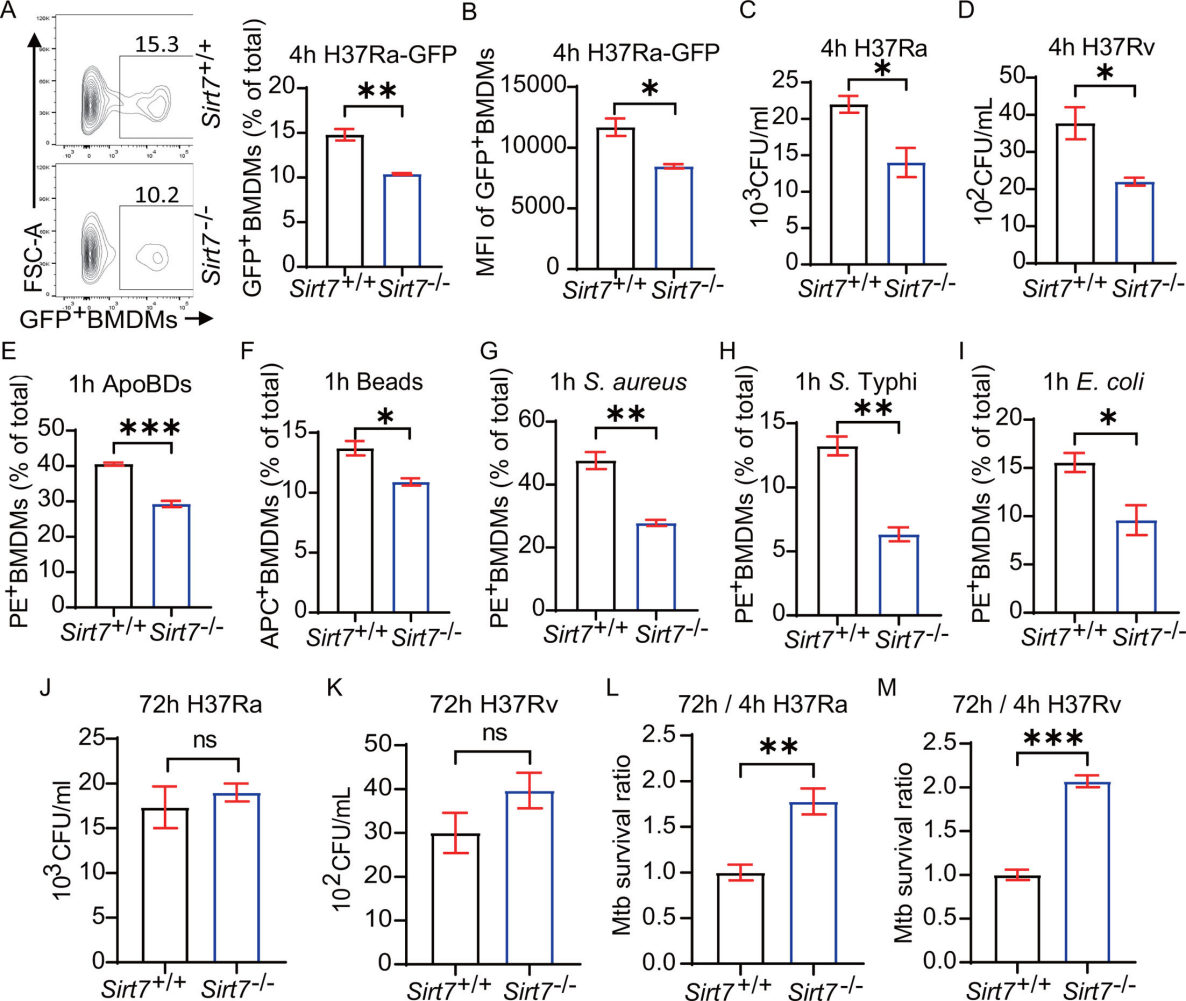
SIRT7 deficiency impairs macrophage phagocytosis and bactericidal capacity. (**A and B**) Flow cytometry analyses and quantification of GFP-H37Ra phagocytosis by *Sirt7^−/−^* BMDMs compared with *Sirt7^+/+^* BMDMs. Histograms illustrate the percentage of macrophages that phagocytosed GFP-H37Ra, with GFP^+^ BMDMs indicating cells that ingested GFP-H37Ra. (**C, D, J, and K**) Intracellular CFU counts of H37Ra or H37Rv assessed 4 h (**C and D**) or 72 hours (**J and K**) post-infection in *Sirt7^−/−^* BMDMs and *Sirt7^+/+^* BMDMs. (E through I) Comparative analysis of the percentage of *Sirt7*^−/−^ and *Sirt7*^+/+^ BMDMs phagocytosing apoptotic bodies, beads, *Staphylococcus aureus*, *Escherichia coli*, and *Salmonella* Typhi. After phagocytosis, BMDMs engulfing beads can be detected by APC fluorescence, while the ingestion of pHrodo red-labeled apoptotic bodies and bacteria can be detected by PE fluorescence. (**L and M**) The survival ratio of H37Ra or H37Rv was evaluated in *Sirt7^−/−^* BMDMs compared to WT controls. Data are presented as means ± SEM, ns, not significant, *P < 0.05, **P < 0.01, ***P < 0.001, as determined by Student’s two-tailed unpaired t-test. Each experiment was independently replicated three times.

The above results prompted us to investigate how SIRT7 enhances host resistance against *Mtb* infection. Initially, we explored whether SIRT7 deficiency impacts macrophage phagocytic activity against Mtb. Employing a GFP-expressing *Mtb* strain, GFP-H37Ra, we discovered that SIRT7 deficiency reduced the *Mtb*-phagocytic capability of BMDMs, as evidenced by both flow cytometry and CFU assays at 4 hours (Fig. 3A through D). Flow cytometry results indicated that SIRT7 deficiency led to a reduced proportion of macrophages capable of phagocytosing H37Ra (Fig. 3A). Moreover, the mean fluorescence intensity (MFI) of GFP^+^ macrophage was reduced in the *Sirt7*^−/−^ BMDMs compared to *Sirt7*^+/+^ BMDMs (Fig. 3B), suggesting a reduced bacterial uptake by these macrophages. Similar results were observed in *Mtb*-infected THP1-derived macrophages treated with si-*SIRT7*, among which the percentage and MFI of GFP^+^ macrophages and the numbers of CFU were significantly lower than the controls group (Fig. S3A through F). These results were further confirmed using the virulent strain, H37Rv. After 4 hours of infection, the intracellular H37Rv level in *Sirt7*^−/−^ BMDMs was significantly reduced compared to that of *Sirt7*^+/+^ BMDMs (Fig. 3D). In addition, SIRT7 deficiency also impaired the phagocytosis of apoptotic bodies, *Staphylococcus aureus, Escherichia coli,* and *Salmonella* Typhi (Fig. 3E through I; Fig. S3I through M). These findings indicate that SIRT7 deficiency impacted the macrophages' phagocytic function.

Given that compromised macrophage phagocytosis is closely associated with increased susceptibility to TB (22, 24, 25), we were prompted to investigate the bacterial killing ability of the macrophages. While the CFUs at 72 hours showed no variance (Fig. 3J and K; Fig. S3G), the bacterial survival ratio—defined as the CFU ratio at 72 hours to that at 4 hours—was significantly higher in *Sirt7*^−/−^ BMDMs and si-*SIRT7* THP1-derived macrophages than their WT counterparts (Fig. 3L and M; Fig. S3H). These findings indicate that SIRT7 deficiency enhances the survival of Mtb within macrophages. In summary, the absence of SIRT7 adversely impacted the macrophages’ phagocytic function and their ability to eliminate *Mtb*.

### SIRT7 induces actin cytoskeletal remodeling

**Fig 4:**
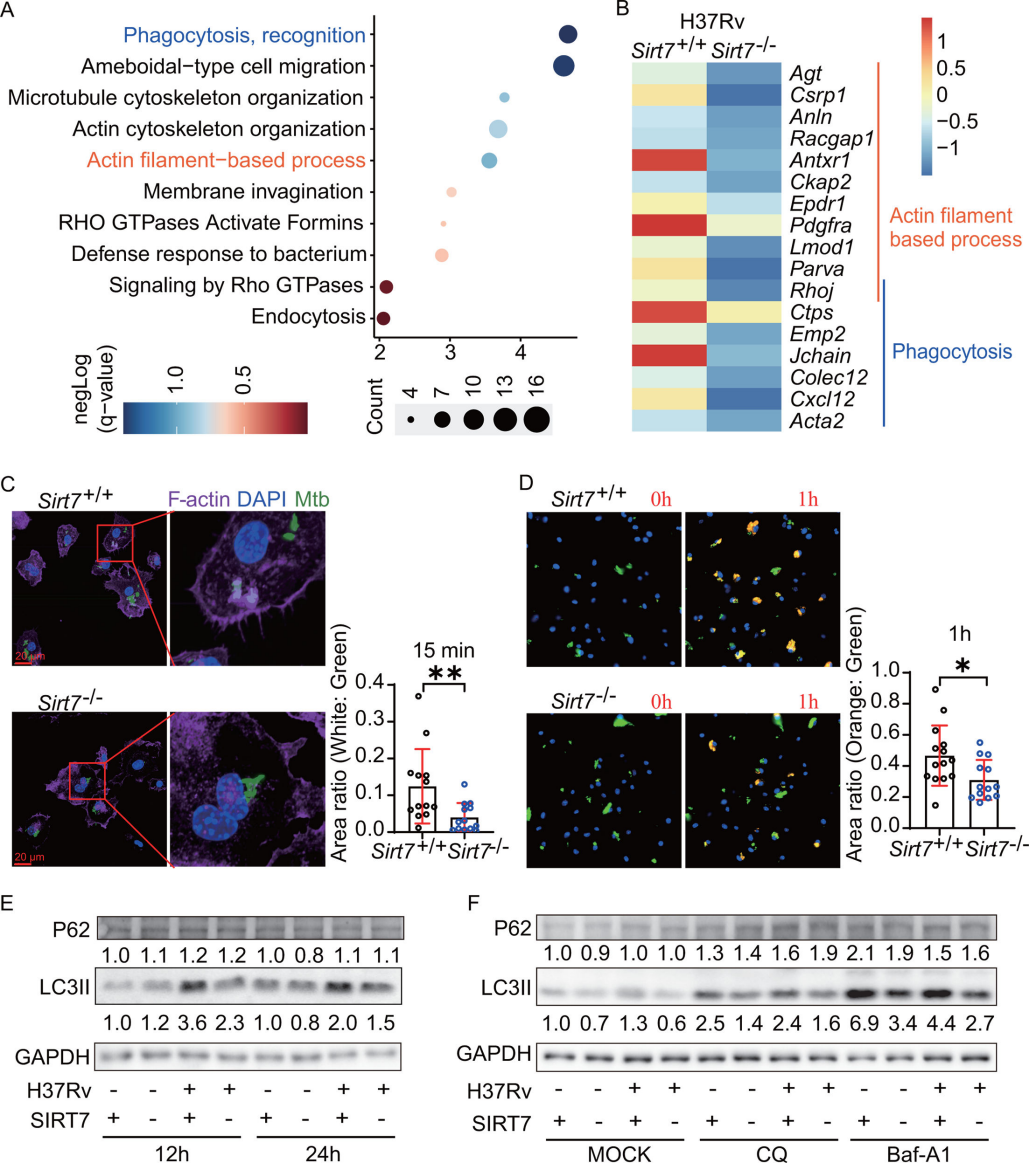
SIRT7 facilitates actin cytoskeletal remodeling during phagocytosis. (**A**) The bubble plot illustrates the GO enrichment of downregulated genes in H37Rv-infected *Sirt7*^−/−^ BMDMs compared to infected *Sirt7*^+/+^ controls. Each bubble, labeled on the y-axis, represents an enriched term. Bubble size corresponds to the count of matched genes in the gene set, and color indicates the enrichment degree [negative-log(q-value)]. Terms are arranged in ascending order based on their negative-log(p-value). (**B**) Heat map comparison of 17 genes associated with actin-filament-based processes (GO:0030029) and phagocytosis (GO:0006910) as shown in (**A**). Z-score-normalized gene expression values for infected *Sirt7*^+/+^ and *Sirt7*^−/−^ groups are displayed. (**C**) Confocal microscopy images of *Sirt7*^−/−^ and *Sirt7*^+/+^ BMDMs infected with GFP-H37Ra (green) at an MOI of 10 for 15 min. Cells were fixed and stained with Phalloidin-iFluor 555 (purple) for F-actin and DAPI (blue) for nuclei. Image J quantified the area of H37Ra-GFP and its co-localization (white) with F-actin in the field of view. The ratio of the white area to the green area indicates the proportion of H37Ra surrounded by the phagocytic cup. (**D**) Time-lapse microscopy was utilized to capture the phagocytosis process of pHrodo Red-labeled GFP-H37Ra (shifting from green to orange upon phagocytosis) by *Sirt7*^−/−^ and *Sirt7*^+/+^ BMDMs. Representative microscopy images illustrate the dynamic phagocytosis of GFP-H37Ra over a 1-hour period, while the accompanying bar graph quantifies the ratio of the orange area (indicative of phagocytosed H37Ra) to the green area (total H37Ra) at the 1-hour time point. (**E and F**) Immunoblot analysis was conducted to assess LC3II and P62 expression levels. (**E**) *Sirt7*^−/−^ and *Sirt7*^+/+^ BMDMs were infected with H37Rv at an MOI of 10, and samples were collected at 12 and 24 hours post-infection for examination. (**F**) To prevent the formation of phagolysosome, 4 hours prior to sampling, CQ (40 µM) or Baf-A1 (100 nM) was added to the corresponding groups. The LC3II and P62 expressions in *Sirt7*^−/−^ and *Sirt7*^+/+^ BMDMs, infected with H37Rv at an MOI of 10 for 24 hours, were analyzed. The expression levels of LC3II, P62, and SIRT7 were quantified relative to GAPDH, as depicted below the respective bands. Data are presented as means ± SEM, *P < 0.05, **P < 0.01, determined by Student’s two-tailed unpaired t-test.

To elucidate the potential mechanisms through which SIRT7 influences macrophage phagocytosis, genome-wide transcriptional analysis was conducted using *Sirt7*^+/+^ and *Sirt7*^−/−^ BMDMs. SIRT7 deficiency altered the expression of 87 genes in *Mtb*-infected BMDMs, with 70 of these genes showing significantly lower expression compared to those in the *Sirt7*^+/+^ BMDMs infected group (Table S1). Analysis of these 70 genes revealed the expression enrichment of genes predominantly involved in regulating phagocytosis, cell migration, actin filament-based processes, and cytoskeleton organization remodeling signals (Fig. 4A and B). Considering that phagocytosis and migration are actin filament-based activities (28, 29), we hypothesized that the impairment of phagocytosis in *Sirt7*^−/−^ BMDMs might be due to cellular actin cytoskeletal remodulation.

The phagocytic cup, defined as a plasma membrane supported by F-actin that encircles pathogens (9), corresponds to the areas of F-actin and GFP-H37Ra colocalization depicted in white in Fig. 4C. These areas are notably diminished in *Sirt7*^−/−^ BMDMs, suggesting that the compromised phagocytic response in these cells is linked to alterations in F-actin cytoskeletal dynamics. Beyond phagocytosis, actin cytoskeletal dynamics are pivotal for cell recruitment and migration during immune responses to bacteria (30). To further elucidate SIRT7’s influence on cytoskeletal dynamics, we conducted a wound-healing assay, which revealed inhibited motility in *Sirt7*^−/−^ BMDMs compared to *Sirt7*^+/+^ BMDMs (Fig. S4A).

Macrophages transport ingested material within acidic phagosomes, and in acidic environments, pHrodo Red dye emits fluorescence (31). To investigate SIRT7’s involvement in macrophage phagocytosis to a greater extent, pHrodo Red dye was used to label GFP-H37Ra, enabling us to differentiate between ingested and adhered GFP-H37Ra using confocal microscopy. As a result of phagocytosis, the color of H37Ra shifted from green to orange. A lower amount of pHrodo-Red-labeled H37Ra color shift was seen in the *Sirt7*^−/−^ group (Fig. 4D; Fig. S4B).

LC3 is pivotal in the maturation processes of both phagosomes and autophagosomes, attaching to the outer membranes of phagosomes through lipidation to create LC3-associated phagosomes (LAPosomes) (32). This process facilitates the transport and fusion of LAPosomes with lysosomes (6). Distinct from autophagosomes, the generation of LAPosomes bypasses the need for the autophagy initiation complex and appears to proceed without the requirement for adaptor proteins like p62 and NDP52 (33). Our research reveals that SIRT7 deficiency markedly reduces LC3II expression in H37Rv-infected BMDMs (Fig. 4E). This reduction is observed even with chloroquine (CQ) and bafilomycin A1 (Baf-A1) treatment (Fig. 4F), indicating a hindrance in LC3II formation attributable to SIRT7 knockout. Importantly, SIRT7 deficiency does not affect P62 expression (Fig. 4E and F), underscoring that SIRT7 deficiency specifically impairs LAPosome formation. Collectively, these findings indicate that the lack of SIRT7 led to a significant decrease in macrophage LC3-associated phagocytic ability through the remodeling of actin cytoskeleton dynamics.

### SIRT7 induces cytoskeletal remodeling *via* RAC1

Previous studies have shown that cytoskeleton rearrangements and phagocytic cup formation are modulated by small GTPase family proteins, which toggle between active GTP-bound forms and inactive GDP-bound states (4, 5, 34). Given our RNA-seq results indicated that SIRT7 deficiency downregulated Rho-GTPase signaling (Fig. 4A and B), we hypothesized that SIRT7 modulates actin-cytoskeleton-dependent cellular phagocytosis *via* Rho-GTPase activity. Specifically, the small GTPases CDC42 and RAC1 have been shown to play roles in multiple macrophage phagocytosis (4, 35). We subsequently determined the activation status of CDC42 and RAC1 in *Mtb*-infected macrophages using an active GTPases pull-down and detection kit, which selectively enriched and detected GTP-bound RAC1/CDC42 GTPases through the p21-activated protein kinase 1 (PAK1) protein-binding domain (36).

**Fig 5:**
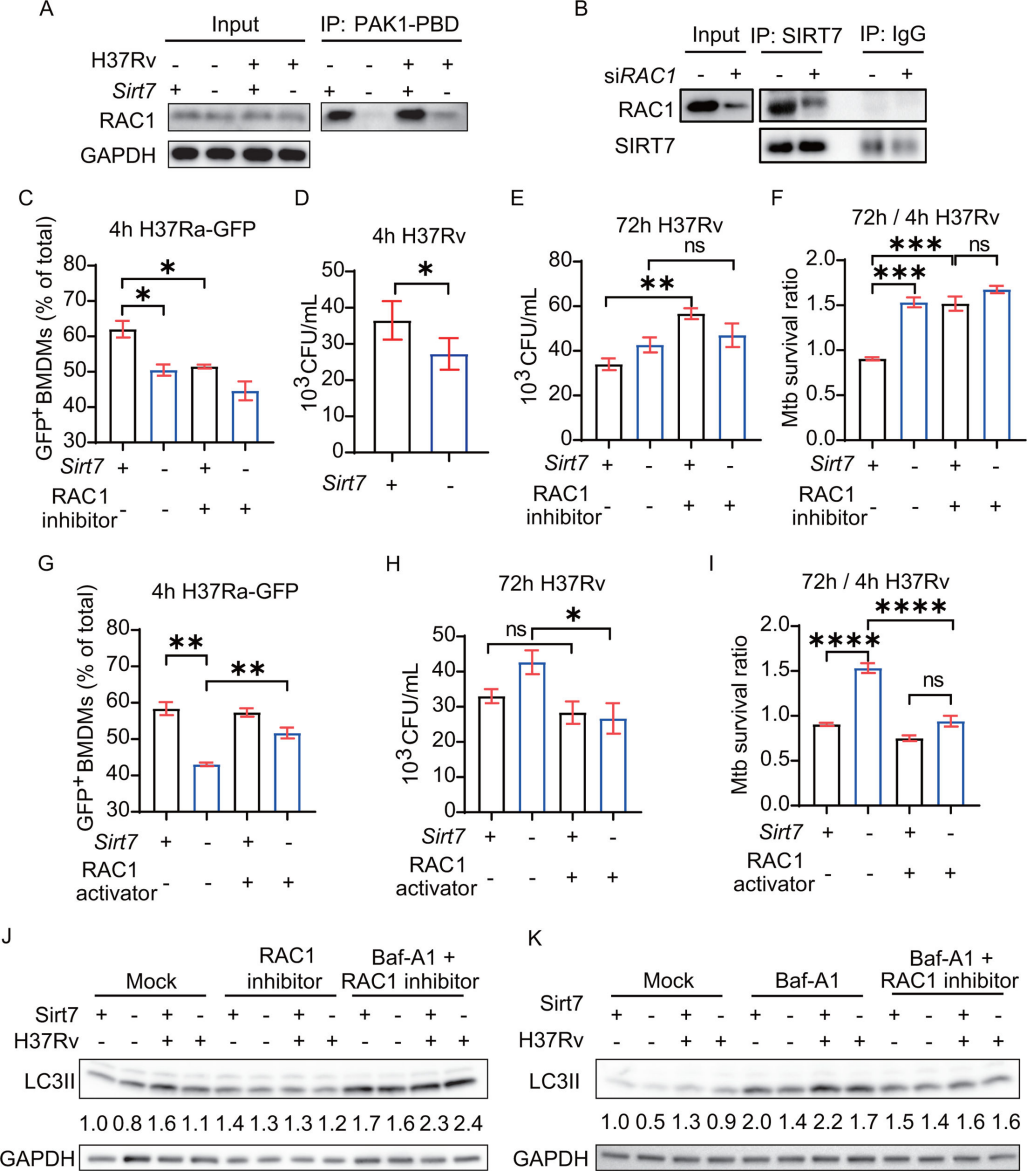
SIRT7 induces cytoskeletal remodeling *via* RAC1. (**A**) RAC1activation in H37Rv-infected *Sirt7*^−/−^ and *Sirt7*^+/+^ BMDMs assayed at 0 min and 30 min post-infection. “Input” represents RAC1 protein levels in whole-cell lysates, while “IP: PAK1-PBD” indicates active RAC1 levels. (**B**) Immunoblot and immunoprecipitation assays illustrating the interaction of SIRT7 and RAC1 in THP1-derived macrophages transfected with si-*RAC1* or normal control (si-NC) siRNA. (**C and G**) Flow cytometry analysis quantifying the phagocytosis of GFP-H37Ra by *Sirt7*^−/−^ and *Sirt7*^+/+^ BMDMs under the conditions of RAC1 inhibitor or activator treatment. GFP^+^ BMDMs represent cells that phagocytosed GFP-H37Ra. (**D**) Intracellular CFU counts of H37Rv at 4 hours post-infection in *Sirt7*^−/−^ and *Sirt7*^+/+^ BMDMs. (**E and H**) Following a 4-hour infection period with H37Rv, macrophages were washed with phosphate-buffered saline (PBS) to eliminate unphagocytosed bacteria. Subsequently, a fresh complete medium was introduced, either containing an RAC1 inhibitor or activator. CFU counts were determined 68 hours later. (**F and I**) The survival ratio of H37Rv was evaluated in *Sirt7^−/−^* BMDMs compared to WT BMDMs, following treatment with either RAC1 inhibitor or activator. (**J and K**) Immunoblot analysis was performed to assess the expression of LC3II in *Sirt7*^−/−^ and *Sirt7*^+/+^ BMDMs treated with an RAC1 inhibitor or Baf-A1. The expression levels of LC3II were quantified and are shown relative to GAPDH, as indicated below the corresponding bands. Data are presented as means ± SEM, *P < 0.05, **P < 0.01, and ***P < 0.001, as determined by one-way ANOVA with Tukey’s multiple comparisons test. Each experiment was independently replicated two to three times.

Although the abundance of total RAC1 in *Sirt7*^−/−^ BMDMs was similar to that in *Sirt7*^+/+^ BMDMs, the amount of active RAC1 was substantially higher in *Sirt7*^+/+^ BMDMs than *Sirt7*^−/−^ BMDMs (Fig. 5A). Activation of CDC42 was not detected in either *Sirt7*^−/−^ or *Sirt7*^+/+^ BMDMs (Fig. S5A). Notably, PAK1-PBD not only interacted with GTP-bound RAC1 but also with SIRT7 in BMDMs (Fig. S5B). To further determine whether SIRT7 directly binds to RAC1, we performed immunoprecipitation assays and confirmed the direct binding of SIRT7 to RAC1 (Fig. 5B). Collectively, these findings indicate that SIRT7 can interact and activate RAC1.

To examine whether the reduced phagocytosis and bactericidal activity observed in *Sirt7*^−/−^ BMDMs resulted from decreased RAC1 activation, we employed an RAC1 activator and inhibitor. The results demonstrated that the RAC1 inhibitor (W56) (37) significantly decreased phagocytosis (Fig. 5C; Fig. S5C and D) and bacterial clearance (Fig. 5D through F) in *Sirt7*^+/+^ BMDMs but not *Sirt7*^−/−^ BMDMs. As anticipated, the impairment in phagocytosis (Fig. 5G; Fig. S5E and F) and bactericidal activity (Fig. 5D, H, and I) resulting from the SIRT7 deficiency in BMDMs could be restored using an RAC1 activator (RAC/CDC42 Activator II) (38). In addition, an RAC1 inhibitor effectively neutralized the differences in LC3II expression observed following SIRT7 knockout (Fig. 5J and K). Baf-A1, by inhibiting autolysosome formation, prevents the degradation of LC3II. The RAC1 inhibitor also neutralized the differences in LC3II expression induced by SIRT7 deficiency under Baf-A1 treatment (Fig. 5J and K). Notably, an RAC1 inhibitor significantly reduced the accumulation of LC3II induced by Baf-A1 (Fig. 5K), underscoring RAC1’s role in the formation of LC3II. Collectively, these results indicate that SIRT7 influences LC3II formation through its regulatory interaction with RAC1. These results demonstrated that the activation of RAC1 is a critical downstream effect of SIRT7 in macrophages in the host defense against infections.

### Overexpression of *Sirt7* enhanced host anti-TB immunity

**Fig 6:**
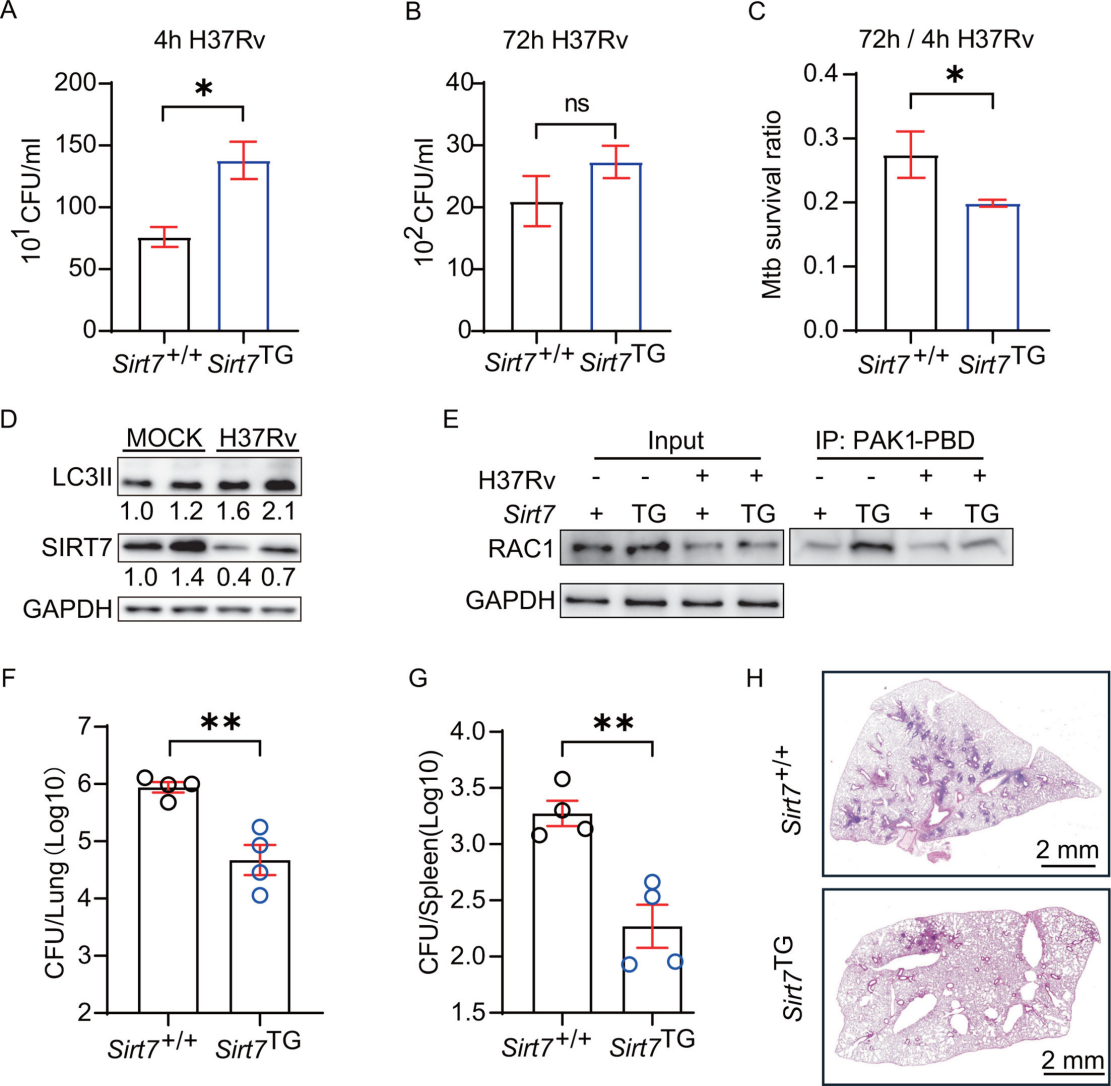
Overexpression of *Sirt7* enhanced host anti-TB immunity. (**A and B**) Intracellular CFU counts of H37Rv at 4 hours and 72 hours post-infection in *Sirt7*^+/+^ and *Sirt7*^TG^ BMDMs. (**C**) The survival ratio of H37Rv was evaluated in *Sirt7*^TG^ BMDMs compared to WT controls. (**D**) LC3II expression was elevated in *Sirt7*^TG^ BMDMs compared to WT controls. (**E**) RAC1 activation in H37Rv-infected *Sirt7*^+/+^ and *Sirt7*^TG^ BMDMs assayed at 0 min and 30 min post-infection, with “Input” indicating total RAC1 and “IP: PAK1-PBD” indicates active RAC1. (**F and G**) Bacterial burden in the lungs and spleens of *Sirt7*^+/+^ and *Sirt7*^TG^ mice was assessed in the tissue homogenates. (**H**) Histopathological analysis of lung sections from *Sirt7*^+/+^ and *Sirt7*^TG^ mice infected with H37Rv. Sections were stained with hematoxylin and eosin, and images were captured using a NanoZoomer digital pathology system. Data are presented as means ± SEM, *P < 0.05, **P < 0.01, and ***P < 0.001 by Student’s two-tailed unpaired t-test. Each experiment was independently replicated two to three times.

We next aimed to discern whether overexpression of SIRT7 would enhance the host immune response to *Mtb* infection. To achieve this, we utilized an inducible *Sirt7* transgenic mouse (*Sirt7*^TG^) (39), wherein *Sirt7* expression could be induced by doxycycline (Dox) at the desired time (Fig. S6A). BMDMs from *Sirt7*^TG^ and *Sirt7*^+/+^ were treated with Dox for 24 hours and then infected with H37Rv. *Sirt7*^TG^ BMDMs exhibited an increased phagocytosis activity, elevated expression of LC3II, and reduced survival ratio of *Mtb*, compared to *Sirt7*^+/+^ BMDMs (Fig. 6A through D). RAC1 activity assays revealed that SIRT7 overexpression also increased RAC1-GTP levels in BMDMs (Fig. 6E), thereby affirming SIRT7’s role in RAC1 activation. Post-infection with *Mtb* (H37Rv), bacterial loads in the lungs and spleens of *Sirt7*^TG^ mice were significantly decreased compared to those in *Sirt7*^+/+^ mice at 4 and 8 weeks post-infection (Fig. 6F and G). Furthermore, enhanced tissue consolidation was observed in the lungs of the *Sirt7*^TG^ mice (Fig. 6H), aligning with the hypothesis that SIRT7 actively modulates host resistance to *Mtb* infections.

## DISCUSSION

The ability of M*tb* to adapt and thrive intracellularly relies on the multiple strategies it uses to alter host innate immune mechanisms. One important aspect of *Mtb* persistence and replication within macrophages has been highlighted by previous studies: interference with phagosome biogenesis (40). In this study, we observed diminished expression of SIRT7 in *Mtb*-infected macrophages and CD14^+^ cells derived from TB patients. We showed that SIRT7-mediated RAC1 activation promoted cytoskeletal remodeling and innate responses during *Mtb* infection. SIRT7 deficiency impaired macrophage LC3-associated phagocytosis and bacterial clearance, increasing host susceptibility to *Mtb* infection. We further showed that SIRT7 enhanced host resistance to *Mtb* infection through the activation of RAC1. Inhibition of RAC1 resulted in reduced bacterial clearance, phenocopying SIRT7 deficiency. These results provide insights into the mechanisms and functions of SIRT7 in regulating host resistance to *Mtb* infection.

The sirtuin (SIRT) family, consisting of NAD+-dependent deacetylases and ADP-ribosyltransferases, plays significant roles in regulating cellular metabolism, stress responses, and longevity (27). Several Sirts have been reported that involved in various cellular processes that impact the immune response to Mtb infection. For example, during Mtb infection, SIRT1 interacts with TAK1 to activate the p65/p38/JNK/ERK signaling pathways, thereby enhancing the host immune response (41). SIRT3 deficiency exacerbates inflammatory responses and mitochondrial dysfunction, leading to impaired host defense and pathological inflammation in mycobacterial infections (42, 43). Conversely, SIRT2 appears to have a less advantageous role; inhibiting SIRT2 in mice leads to a lower bacterial load, milder disease pathology, and enhanced Mtb-specific immune responses (44). Although SIRT7 is involved in various biological functions and its dysregulation is linked to several human diseases (27, 45), its role in bacterial infections, including Mtb, remains largely unexplored. Here, we found a direct association between SIRT7 and TB, in that we observed that SIRT7 expression was specifically downregulated in monocytes from TB patients compared to those from healthy individuals. A previous study reported the downregulation of SIRT7 expression in *Mtb*-infected macrophages and highlighted its role in promoting *Mtb* clearance in Raw 264.7 macrophages (46). Aligning with their findings, we also observed the downregulation of SIRT7 expression in macrophages during *Mtb* infection. Despite this, the authors of the previous study found that the enhanced macrophage bactericidal activity induced by SIRT7 was dependent on Nos2 expression, which differed from our present study, in which SIRT7 was shown to facilitate RAC1 activation to enhance phagocytosis and bacterial clearance. In our study, we did not detect any difference in iNOS at either mRNA or protein levels between *Sirt7*^+/+^ and *Sirt7*^−/−^ BMDMs (Fig. S6B and C). These contradictory results can be explained by the different cellular and experimental settings in which the effects of SIRT7 have been monitored. In their study, they used the highly proliferative cell lines RAW 264.7 and the pan-sirtuin family inhibitor, nicotinamide, which has been reported to inhibit all sirtuins (46, 47), whereas we obtained most of our results using primarily *Sirt7*^−/−^ BMDMs. In addition, our results indicate that SIRT7 deficiency does not affect apoptosis, cytosolic reactive oxygen species (cROS), or mitochondrial reactive oxygen species (mROS) in BMDMs (Fig. S6D through F).

Macrophage phagocytosis is a hallmark of antibacterial host defenses, and actin filament network-mediated phagocytosis plays an important role in effective TB control. Previous studies have shown that pathogenic mycobacteria induce dysfunction in the host cells’ actin filament networks, thwarting the macrophage-mediated elimination of *Mtb* (7, 14, 15). Impairment of macrophage phagocytic ability has been observed in elderly, diabetic, and HIV-infected individuals, who are highly predisposed to TB (17, 48, 49). This impairment of macrophage function has been strongly linked to an increase in susceptibility to TB (22, 24, 25). Consistent with this, our results revealed a deficiency in SIRT7 that resulted in reduced macrophage phagocytosis and increased host susceptibility to TB. In addition, our observations suggest that the influence of SIRT7 on macrophage phagocytosis is not limited to *Mtb*. SIRT7 deficiency also impaired the phagocytosis of apoptotic bodies, *Staphylococcus aureus, Escherichia coli,* and *Salmonella* Typhi. In this regard, SIRT7 might influence actin cytoskeletal rearrangement rather than the activity of receptors related to phagocytic recognition. Of note, our transcriptome analysis revealed that *SIRT7* deficiency resulted in the downregulation of actin filament-based process signals. Given the central role of cytoskeletal rearrangements in the formation of the phagocytic cup, the suppression of these activities in *Sirt7*^−/−^ BMDMs suggests that SIRT7 controls macrophage phagocytosis by regulating the actin cytoskeleton. Concurrently, it was observed that SIRT7 deficiency reduced LC3II protein levels without impacting P62 expression. LC3 associates with phagosome membranes, enhancing their cytoskeletal transport and lysosomal fusion. Phagocytosis, unlike autophagy, does not necessitate adaptor proteins like P62 for targeting degradative cargo. Thus, it is posited that SIRT7 deficiency disrupts the formation of LC3-associated phagosomes, impairing macrophages’ capacity to eliminate *Mtb*.

Rho-GTPase is widely recognized as a crucial molecule involved in regulating actin cytoskeleton dynamics (4, 5). Prior studies established that macrophages deficient in RAC1 exhibit compromised phagosome maturation (50). Pathogenic bacteria secrete various virulence determinants that alter phagosome biogenesis, facilitating their survival within cells. For example, *Vibrio cholerae* effector, a Rho GTPase inactivation domain, was shown to bind to RAC1, trapping it at the cell membrane and inhibiting its activation (51). *Mtb* nucleoside diphosphate kinase binding to and inactivation of RAC1 in macrophages resulted in their increased susceptibility to *Mtb* infection (52). Therefore, the proper activation of RAC1 is a critical protective mechanism against bacterial infection. While the molecular events downstream of RAC1 that enable macrophage phagocytosis have been revealed in detail, how the activation of RAC1 is regulated in macrophages during *Mtb* infection is far from understood. A previous study showed that CYFIP-related RAC1-interacting protein negatively regulates RAC1 signaling to attenuate phagocytosis and cell migration, conferring host resistance to *Salmonella* infection (53). Another study showed that bacterial infection triggers the assembly of the Piezo1-TLR4 complex to remodel the F-actin organization and enhance phagocytosis and bacterial clearance *via* the CaMKII-Mst1/2-Rac axis (54). Our study identified a previously uncharacterized role for SIRT7 in mediating RAC1 activation. We found that the absence of SIRT7 resulted in cytoskeleton remodeling through the inhibition of RAC1 activity. Apart from its protective effects against *Mtb* phagocytosis, SIRT7 also suppresses intracellular *Mtb* growth. These two distinct biological effects have similar mechanisms that involve the activation of RAC1, which not only affects the initiation of phagocytosis but also regulates fusion events between early endosomes and phagosomes.

RAC1 activation is tightly regulated by guanine-nucleotide exchange factors (GEFs) and GTPase-activating proteins. In addition, recent findings showed that the activity of RAC1 is regulated by acetylation modification (55). Most biological functions of SIRT7 can be attributed to its deacetylase activity. SIRT7 deacetylates histone or non-histone proteins to regulate chromatin architecture homeostasis and metabolism (56). Considering the deacetylase activity of SIRT7, we propose that SIRT7 deacetylates RAC1 to activate it. Despite assessing both *Mtb*-infected and uninfected BMDMs, we did not detect any acetylation of RAC1 (Fig. S7). However, we cannot rule out the possibility that SIRT7 facilitates RAC1 activation through GEFs or other modifications such as desuccinylation, deglutarylation, and defatty-acylation (57–59). Further studies, specifically those investigating the specific mechanism of SIRT7-mediated RAC1 activation, are highly encouraged to identify new therapeutic targets in TB.

Neutrophils play a critical role in the immune response to pulmonary Mtb infection, as their recruitment is a key aspect of TB pathology (60). Previous studies have demonstrated that SIRT7 deficiency significantly increases neutrophil counts in the blood of uninfected mice without affecting other cell types (61). In our murine TB model, we observed that both the proportion and absolute number of neutrophils were markedly elevated in the lungs of *Sirt7*^−/−^ mice. This increase in neutrophils was accompanied by elevated levels of the chemokines CXCL1 and CXCL2, which are crucial for neutrophil recruitment (62, 63). These findings suggest that the heightened neutrophil counts in Mtb-infected *Sirt7*^−/−^ mice could be due to increased neutrophil recruitment driven by these chemokines. However, the mechanisms by which SIRT7 influences neutrophil recruitment in the lungs during Mtb infection warrant further investigation.

In conclusion, although there have been extensive studies on the role of SIRT7 in physiological processes, its contribution to *Mtb* pathogenesis remained largely unknown prior to this investigation. We have demonstrated that SIRT7 restricts *Mtb* infection at different stages of TB in a manner consistently reliant on RAC1 signaling. Beyond *Mtb*, we found that SIRT7 deficiency broadly affected macrophage phagocytic activity. Our findings have expanded our understanding of the biological effects of SIRT7, such as the activation of GTPase effector functions in macrophages. In addition, phagocytes play a role in facilitating systemic infections of *Mtb* (64). We therefore propose the hypothesis that SIRT7’s capacity to regulate the cytoskeleton remodels phagocytes and offers another vital defense mechanism that restricts bacterial dissemination.

## MATERIALS AND METHODS

### Clinical sample collection

All TB patient samples and healthy controls utilized in this study were obtained from Shenzhen Third Hospital and Shenzhen People’s Hospital. The study included 17 HCs and 15 cases of active pulmonary TB. The diagnosis of active pulmonary TB was confirmed based on clinical symptoms, chest X-rays, acid-fast *Bacilli* staining, microscopy of sputum and/or bronchoalveolar lavage fluid, *Mtb* culture, and responses to anti-TB drugs. Notably, these patients did not have comorbidities such as diabetes or HIV/AIDS. The control group consisted of individuals with no clinical history of TB and normal chest X-rays. Active pulmonary TB was diagnosed by culturing pleural effusion and/or biopsy specimens or observing granulomatous inflammation in pleural biopsy tissue, as previously described (65). Heparinized whole-blood samples were collected from all participants by venipuncture, and peripheral blood mononuclear cells (PBMCs) were obtained through whole-blood gradient separation, as previously described (66). The separated PBMCs were immediately used for flow cytometry.

### Reagents

The following antibodies were used: Anti-SIRT7 (Santa Cruz Biotechnology, #sc-365344), anti-RAC1 (Abcam, #ab155938), anti-RAC1 (cytoskeleton, #ARC03), anti-LC3II (R&D, #MAB85581), anti-P62 (Abcam, #ab109012), Mouse mAb IgG1 Isotype (cell signaling technology, # 5415), Rat anti-Mouse IgG1 Secondary (Invitrogen, #11–4015-82), PE/Cyanine7 anti-human CD3 (Biolegend, #300316), APC anti-human CD14 (Biolegend, #325608), APC/Cyanine7 anti-human CD19 (Biolegend, #302218), anti-GAPDH (Abcam, #ab9485), Acetylated-Lysine Antibody (CST, #9441), Zombie Aqua (Biolegend, #423102), PE/Cyanine7 anti-mouse CD3 (Biolegend, #100220), APC anti-mouse CD45 (Biolegend, #147708), PE anti-mouse Ly-6G (Biolegend, #127608), Brilliant Violet 605 anti-mouse Ly-6C (Biolegend, #128036), Alexa Fluor 488 anti-mouse CD170 (Biolegend, # 155524), PE/Cyanine5 anti-mouse/human CD11b (Biolegend, #101210), and APC/Cyanine7 anti-mouse CD19 (Biolegend, #115530). The following chemicals were used: RAC1 Inhibitor W56 (Medchemexpress, #HY-P1382), RAC/CDC42 Activator II (cytoskeleton, #CN02), pHrodo Red succinimidyl (NHS) ester (Invitrogen, #P36600), Mouse Intracellular (nuclear/transcription factor) Protein Flow Cytometry Workflow Kit (Invitrogen, # A53017), pHrodo Red succinimidyl ester (Invitrogen, #P36600), Active Rac1 Pull-Down and Detection Kit (Thermo Scientific, #16118), SPHEROTM Fluorescent Particles (Spherotech, #FL-6068), Bafilomycin A1 (MCE, #HY-100558), Chloroquine (MCE, # HY-17589A), fluorescein isothiocyanate (FITC) Annexin V Apoptosis Detection Kit with PI (BioLegend, #640914), MitoSOX Mitochondrial Superoxide Indicators (Invitrogen, # M36008), H2DCF-DA (Invitrogen, # D399), Mouse CXCL1 ELISA Kit (HUABIO, # EM0017), Mouse CXCL2 ELISA Kit (HUABIO, # EM0018).

### Mice and cell culture

*Sirt7*^−/−^ mice and *Sirt7*^TG^ mice were obtained from Baohua Liu at Shenzhen University Medical School. *Sirt7*^−/−^ mice were backcrossed for at least three generations to separate potential off-target deletions (67). *Sirt7*^TG^ mice were generated and bred as previously described (39). All mouse work was conducted in accordance with the guidelines set by the Institutional Animal Committee of Shenzhen University School of Medicine (Approval No.:IACUC-202300031). Human monocytic THP1 cells (TIB-202, ATCC) were cultured at a density of 4 × 10^5^ cells/mL in 6-well or 12-well plates (Costar) and treated with 40 ng/mL Phorbol 12-myristate 13-acetate (PMA) (Sigma-Aldrich) for 48 hours to induce differentiation into macrophages. BMDMs were isolated from *Sirt7*^+/+^, *Sirt7*^−/−^, or *Sirt7*^TG^ mice as previously described (68). Bone marrow cells were cultured in DMEM supplemented with 20% L929 cell-conditioned medium, 1 mM sodium pyruvate, 2 mM L-glutamine, and 10% fetal bovine serum for 7 days. The culture and induction process were carried out at 37°C in a humidified incubator with 5% CO_2_. PMA-differentiated THP1 macrophages and BMDMs were kept in a fresh, pre-warmed culture medium until further use.

### Bacterial strains and culture

The mycobacterial strains utilized in this investigation comprise the virulent *Mtb* strain H37Rv, the attenuated Mtb strain H37Ra, and H37Ra expressing the GFP reporter. Cultivation was performed in Middlebrook 7H9 broth supplemented with 0.2% glycerol, 0.25% Tween-80, and 10% OADC until a logarithmic growth phase was achieved (OD_595_ ~0.3–0.4). Bacterial dilutions were plated on Middlebrook 7H10 agar and incubated at 37°C for 21 days to count colony-forming units (CFU) for determining bacterial loads for experiments. Tween-80 was used to disaggregate bacterial clumps, complemented by ultrasonication for enhanced dispersion. To evaluate bacterial loads in mouse lung homogenates and cell lysates, samples were serially diluted, plated on Middlebrook 7H10 agar, and incubated for 21 days for CFU enumeration.

### Murine TB model

All mouse work was conducted in accordance with the guidelines set by the Institutional Animal Committee of Shenzhen University School of Medicine. *Sirt7*^–/–^, *Sirt7*^+/+^, and *Sirt7*^TG^ mice aged 6–8 weeks were randomly assigned to separate cages and infected with approximately 100–200 CFU of the H37Rv strain using the Glas-Col inhalation exposure system, as previously described (69). In the case of *Sirt7*^TG^ mice, 1 mg/mL of Dox was administered orally *via* drinking water 7 days before infection to induce *Sirt7* overexpression. During the infection period, Dox was administered in the drinking water only during the first 3 days of each week. Three to four animals were sacrificed on day 1 to determine the number of bacteria implanted in the lungs. Mice were sacrificed at 28 or 56 days post-infection. Lungs were aseptically excised and homogenized, followed by two to three 10-fold serial dilutions and subsequently cultured on 7H11-OADC agar plates. CFUs were enumerated after 2–3 weeks of incubation at 37°C in a 5% CO_2_ atmosphere. Lung tissue fragments were fixed in 10% formalin, embedded in paraffin, and sectioned into 4 mm slices. Tissue sections were stained with hematoxylin and eosin (H&E) according to standard protocols. Images of the entire microscope slides were captured using the NanoZoomer digital pathology system (Hamamatsu Photonics), as previously described (69). Lung tissue was digested and ground to obtain lung cells, which were counted and then subjected to standard flow cytometry to measure the numbers of different immune cells. CXCL1 and CXCL2 from lung homogenates were examined using the standard sandwich enzyme-linked immunosorbent assay (ELISA) protocol.

### Phagocytosis assays

Cell counting was performed using the Luna II system (BioCat, Germany). A total of 1 × 10^5^ THP1 cells or 2.5 × 10^5^ BMDMs were seeded into 12-well plates and treated as per the respective experimental protocols. To assess phagocytosis and survival ratio using the CFU method, PMA-differentiated THP1 macrophages were infected with H37Ra or H37Rv (MOI = 10) for 4 hours, while BMDMs were infected for 4 hours, followed by three washes with phosphate-buffered saline (PBS) to remove uningested *Mtb*. Subsequently, cells were lysed with 0.1% SDS, and serial dilutions were plated onto 7H10 agar plates. CFUs were counted after 2–4 weeks of incubation at 37°C. Similarly, additional cells infected under the same conditions were washed three times with PBS, cultured for an additional 72 hours, and then subjected CFU counting. For flow cytometry-based phagocytosis detection, THP1 macrophages were infected with GFP-H37Ra (MOI = 10) for 4 hours, while BMDMs were infected for 4 hours, followed by three washes with PBS. Cells were incubated on ice for 5 min in PBS containing 1 mM EDTA, then collected, and uninfected cells were used as negative controls.

For confocal-microscopy-based phagocytosis detection, GFP-H37Ra was labeled with pHrodo Red succinimidyl ester (NHS) following the manufacturer’s instructions. Time-lapse microscopy was conducted using a Nikon Eclipse Ti E inverted microscope system (Nikon, Japan), equipped with a stage incubation chamber (Okolab, Italy) and maintained at 37°C, 7.5% CO_2_, and 90% humidity. Images were captured every 3 min for 2 hours using a 20× CFI60 objective lens and NIS Elements software. In each experiment, a minimum of 30 cells were randomly selected for tracking. Upon macrophage ingestion of GFP-H37Ra labeled with pHrodo Red succinimidyl ester, the acidic phagosome environment triggered the pHrodo Red succinimidyl ester to cause GFP-H37Ra, which emits green light, to also emit red light detectable by confocal microscopy. The green area, representing the total amount of H37Ra in each field of view, was calculated using CellProfiler software. Subsequently, the red area, indicative of the amount of H37Ra ingested by macrophages, was calculated. The ratio of the red area to the green area was utilized to represent the phagocytosis rate of H37Ra.

### Phagocytic cup assays

BMDMs (5 × 10^4^ cells) were seeded onto glass coverslips in 12-well plates and allowed to adhere overnight. Subsequently, the medium was substituted with DMEM complete medium containing H37Ra-GFP (10 MOI) and incubated for 15 min. Floating H37Ra-GFP was removed using 1 × PBS. Cells were then subsequently fixed at room temperature for 10–30 min using PBS containing 3%–4% formaldehyde. Following a PBS wash, the cell membranes were permeabilized with 0.1% Triton X-100. After an additional wash, F-actin was stained with Phalloidin-iFluor 555, while cell nuclei were stained with DAPI. After a final PBS wash, the cells were mounted and examined under a confocal microscope. Considering the cells’ three-dimensional structure, multi-layer images were taken from the base to the top of each cell with a Step Size set at 0.5 µm and a scanning height of approximately 20 µm. Scanned images from different planes were merged into a single image for analyzing the co-localization of F-actin and H37Ra-GFP. The Image J software was utilized to calculate the area of H37Ra-GFP and the co-localized area of F-actin and H37Ra-GFP in the field of view. The phagocytic cup formation rate was calculated by dividing the co-localized area by the area of H37Ra-GFP. According to the manufacturer’s instructions, use pHrodo Red succinimidyl ester to label apoptotic bodies, *Staphylococcus aureus*, *Escherichia coli*, and *Salmonella* Typhi, and then assess the phagocytic activity of BMDMs against them. The beads used are 6.3 µm SPHERO Fluorescent Particles, which come with a stable APC (Allophycocyanin) color.

### Wound healing assay

Seed the BMDMs (1 × 10^6^ cells/mL) in a 12-well plate and incubate overnight to allow for adequate cell adhesion. Create a straight-line scratch using the tip of a 200 µL pipette tip, ensuring that the tip remains vertical to the bottom of the well. It is crucial to maintain contact between the pipette tip and the well bottom to ensure consistent removal of the cell layer. Gently wash the cell monolayer to remove detached cells. Replenish the wells with fresh medium. Initially, capture images of the scratch using a phase-contrast microscope at 10× magnification. Place the plate back in the incubator. Perform follow-up imaging at 12 and 24 hours to assess cell migration and wound closure.

### Flow cytometry procedure

SIRT7 expression in PBMCs from healthy individuals and TB patients was assessed *via* flow cytometry. The procedure adhered to the steps outlined in the Mouse Intracellular (nuclear/transcription factor) Protein Flow Cytometry Workflow Kit (Invitrogen, # A53017). Briefly, red blood cells were lysed using a lysis solution (BD Bioscience, #347691), and Fc receptors were blocked for 10–20 min (BD Bioscience, #564220), followed by staining with anti-CD14 (Biolegend, #325608) and anti-CD3 antibodies (Biolegend, #300316). Subsequently, cells were washed with a flow cytometry staining buffer, then fixed and permeabilized. Anti-SIRT7 antibody (Santa Cruz Biotechnology, #sc-365344), resuspended in permeabilization wash buffer, was added and incubated for 50 min at 4°C, with a parallel group co-incubated with an isotype control. Following a wash with 1× permeabilization wash buffer, secondary antibodies were added and incubated for 30 min at 4°C. After washing, cells were resuspended in a flow cytometry staining buffer, collected *via* a flow cytometer, and data were analyzed using FACSDiva software (BD Biosciences).

### siRNAs and transfection

For siRNA transfection, 10 nM *SIRT7* siRNA (5′-CUCACCGUAUUUCUACUACUA-3′) and *RAC1* siRNA (5′-UGAUGCAGGACUCACAAGG-3′) were introduced into cells using Lipofectamine RNAiMAX (Invitrogen) while adhering to the manufacturer’s protocol and as previously described (69). Scrambled siRNA was used for the negative control cells. Knockdown efficiency was assessed by western blotting 36–48 hours post-transfection.

### RNA extraction and quantitative reverse transcription PCR

Total RNA from treated BMDMs was extracted using the RNeasy Kit (Omega, USA), following the manufacturer’s instructions. DNA contamination was removed, and reverse transcription was conducted using HiScript II Q RT SuperMix for qPCR (Vazyme, China). Target gene expression was analyzed using the 7500 Fast Real-Time PCR System (Applied Biosystems, Thermo Fisher Scientific Inc., USA) and SYBR Green Real-Time PCR Master Mix (Bimake, USA). Relative mRNA expression of target genes was normalized to the reference gene, glyceraldehyde 3-phosphate dehydrogenase (*Gpdh*), and calculated using the 2^−∆∆Ct^ method. Primers, sourced from PrimerBank, were as follows: m-*Sirt7* (forward primer: 5′- AGCATCACCCGTTTGCATGA-3′; reverse primer: 5′-GGCAGTACGCTCAGTCACAT-3′), m-*Gapdh* (forward primer: 5′- AGGTCGGTGTGAACGGATTTG-3′; reverse primer: 5′-TCATC- TGTAGACCATGTAGTTGAGGTCA-3′), h-*SIRT7* (Forward primer: 5′- AGAAGCGTTAGTGCTGCCG-3′; Reverse primer: 5′- GAGCCCGTCACAGTTCTGAG-3′), and h-*GAPDH* (Forward primer: 5′- CTGGGCTACACTGAGCACC-3′; Reverse primer: 5′- AAGTGGTCGTTGAGGGCAATG-3′).

### Transcriptome sequencing analysis

Total RNA was extracted from BMDMs of *Sirt7*^+/+^ or *Sirt7*^−/−^, either uninfected or infected with H37Rv, at 12 hours post-infection using the mirVana Isolation Kit (Life Technologies). BMDMs were derived from three mice per group, aged 8–12 weeks. Total RNA from each sample was also extracted using TRIzol Reagent, RNeasy Mini Kit (Qiagen). Each sample’s total RNA was quantified and qualified using an Agilent 2100/2200 Bioanalyzer (Agilent Technologies, Palo Alto, CA, USA) and NanoDrop (Thermo Fisher Scientific Inc.). Then, 1 µg of total RNA was utilized for subsequent library preparation. Following library construction, purification, detection, and quantification, sequencing data were quality-controlled using FastQC and subsequently filtered with Cutadapt. Short reads were aligned utilizing Hisat2 (v2.0.1) software, while gene expression was calculated using the fragments per kilo bases per million reads method (70) *via* the Htseq software (V0.6.1). Differential gene expression analysis was conducted utilizing the DESeq2 package from Bioconductor, while Gene Ontology (GO) enrichment analysis was performed with GOseq (71). The RNA-seq data generated in this study have been deposited in the National Center for Biotechnology Information’s Gene Expression Omnibus (NCBI GEO), accessible through GEO Series accession number GSE245205.

### Western blotting

After treatment with siRNA and/or H37Ra/H37Rv (MOI = 10), differentiated THP1 cells and BMDMs were washed with 1× PBS and lysed using lysis buffer (P0013, Beyotime, Shanghai, China). Protein concentration was determined using a Bicinchoninic Acid Assay Kit (Beyotime, Shanghai, China). Equal amounts of protein from each sample were then separated using SDS-PAGE gel electrophoresis at appropriate concentrations. Proteins were subsequently transferred onto a PVDF membrane (Merck/Millipore) and blocked at room temperature in PBST containing 1× PBS, 0.1% Tween 20, and 5% nonfat milk powder (GBCBIO Technologic Inc., China) for 1 hour. The blocked membrane, after being washed twice with PBST, was incubated overnight at 4°C with antibodies specific to the proteins under investigation. The membrane, after three washes with PBST (5 min each with shaking), was incubated at room temperature for 1 hour with an appropriate secondary antibody conjugated with horseradish peroxidase (Abcam, USA, 1:10,000). Following a wash with PBST, the membrane was visualized using SuperSignal West Pico PLUS Solution (Thermo Fisher Scientific Inc.) on a MiniChemi imaging system (Sagecreation, China). Signals were quantified using densitometry with ImageJ. Background staining was minimized using the ImageJ plugin Rolling Ball Background Subtraction. The average intensities of the bands of interest were normalized to their respective loading controls.

### RAC1 activation assay

RAC1 activation was assessed using the Active RAC1 Pull-Down and Detection Kit as indicated by the manufacturer (Thermo Scientific, #16118). Briefly, BMDMs were co-incubated with H37Rv for 30 min and washed twice with PBS at 4°C. The entire RAC1 activity detection process was conducted at a low temperature (4°C) while adhering strictly to the manufacturer’s instructions.

### Immunoprecipitation

Post-treatment, cell lysates were harvested using NETN buffer, which contained 150 mM NaCl, 20 mM Tris-HCl (pH 7.5), 1 mM EDTA, 0.5% Nonidet P-40, and a protease inhibitor mixture (Roche). Antibody and control IgG (1 µg) were used for the immunoprecipitation of 200 µg of total lysate, and incubated overnight at 4°C. The precipitates were washed three times with NETN buffer and quantified on a Qubit 4.0 Fluorometer using the Qubit Protein Assay Kit (Thermo Fisher Scientific). Samples were separated via SDS-PAGE and transferred onto a PVDF membrane (Merck Millipore). Membranes were blocked with 5% non-fat milk in PBST (PBS with 0.05% Tween-20) for 2 hours at room temperature, followed by overnight incubation at 4°C with primary antibodies against the target proteins. After washing with PBST, the membrane was incubated for 1 hour at room temperature with horseradish-peroxidase-conjugated secondary antibodies, and visualized with an ECL detection reagent (Thermo Fisher Scientific).

### Apoptosis assay

BMDMs from *Sirt7*^+/+^ and *Sirt7*^−/−^ mice were either left uninfected or infected with H37Rv (10 MOI) for 48 hours. To assess apoptosis, cells were stained using a FITC Annexin V Apoptosis Detection Kit. Following two washes with cold PBS, the cells were resuspended in 5 µL of Annexin V-FITC and 195 µL of binding buffer. The suspension was incubated on ice for 10 to 20 min in the dark. Subsequently, cells were stained with 10 µL of propidium iodide. Apoptosis was then analyzed by flow cytometry using a FACSAria II flow cytometer (BD, USA). Data processing was performed with FlowJo software version 10 (BD Biosciences, USA).

### ROS measurement

BMDMs from *Sirt7*^+/+^ and *Sirt7*^−/−^ mice were either left uninfected or infected with H37Rv (10 MOI) for 6 hours. The cells were washed three times in a prewarmed serum-free RPMI 1640 medium. Endogenous cROS and mROS levels were determined by incubating the cells with 10 µM 2,7-diacetate dichlorofluorescein (H2DCF-DA) and 10 µM MitoSOX Red Mitochondrial Superoxide Indicator, respectively, for 20 min at 37°C in the dark. After incubation, ROS levels were determined by flow cytometric measurement of the MFI using a FACSAria II flow cytometer, and data were analyzed with FlowJo software version 10.

### Statistical analyses

Statistical analyses were conducted utilizing GraphPad Prism version 8 (GraphPad Software Inc.). The statistical significance of differences between groups was ascertained using a Student’s unpaired t-test, one-way ANOVA with Tukey’s post hoc test, two-way ANOVA with Sidak’s multiple comparisons test, or two-way ANOVA with Bonferroni’s post hoc test. Differences were considered to be significant at *P* < 0.05. Details of the statistical analyses for experiments are provided in the figure legends.

## SUPPLEMENTAL MATERIAL

Supplemental Figures mbio.00756-24-s0001.docx Fig. S1 to S7.

Supplemental Tables mbio.00756-24-s0002.docx Tables S1 to S3.

ASM does not own the copyrights to Supplemental Material that may be linked to, or accessed through, an article. The authors have granted ASM a non-exclusive, world-wide license to publish the Supplemental Material files. Please contact the corresponding author directly for reuse.
