# Supplementary material for: SIRT7 remodels the cytoskeleton via RAC1 to enhance host resistance to Mycobacterium tuberculosis
Source: mBio. 2024 Sep 17;15(10):e00756-24. doi: 10.1128/mbio.00756-24 (PMC11481912; doi:10.1128/mbio.00756-24)
Supplement: Supplemental Figures — Fig. S1 to S7. [file mbio.00756-24-s0001.docx]

**Supplemental Materials for**

# SIRT7 remodels the cytoskeleton via RAC1 to enhance host resistance to *Mycobacterium tuberculosis*

Fuxiang Li, Ximeng Zhang, Jinjin Xu, Yue Zhang, Guo Li, Xirui Yang, Guofang Deng, Youchao Dai, Baohua Liu, Christian Kosan, Xinchun Chen, Yi Cai

**This file includes Figure S1 to S7.**


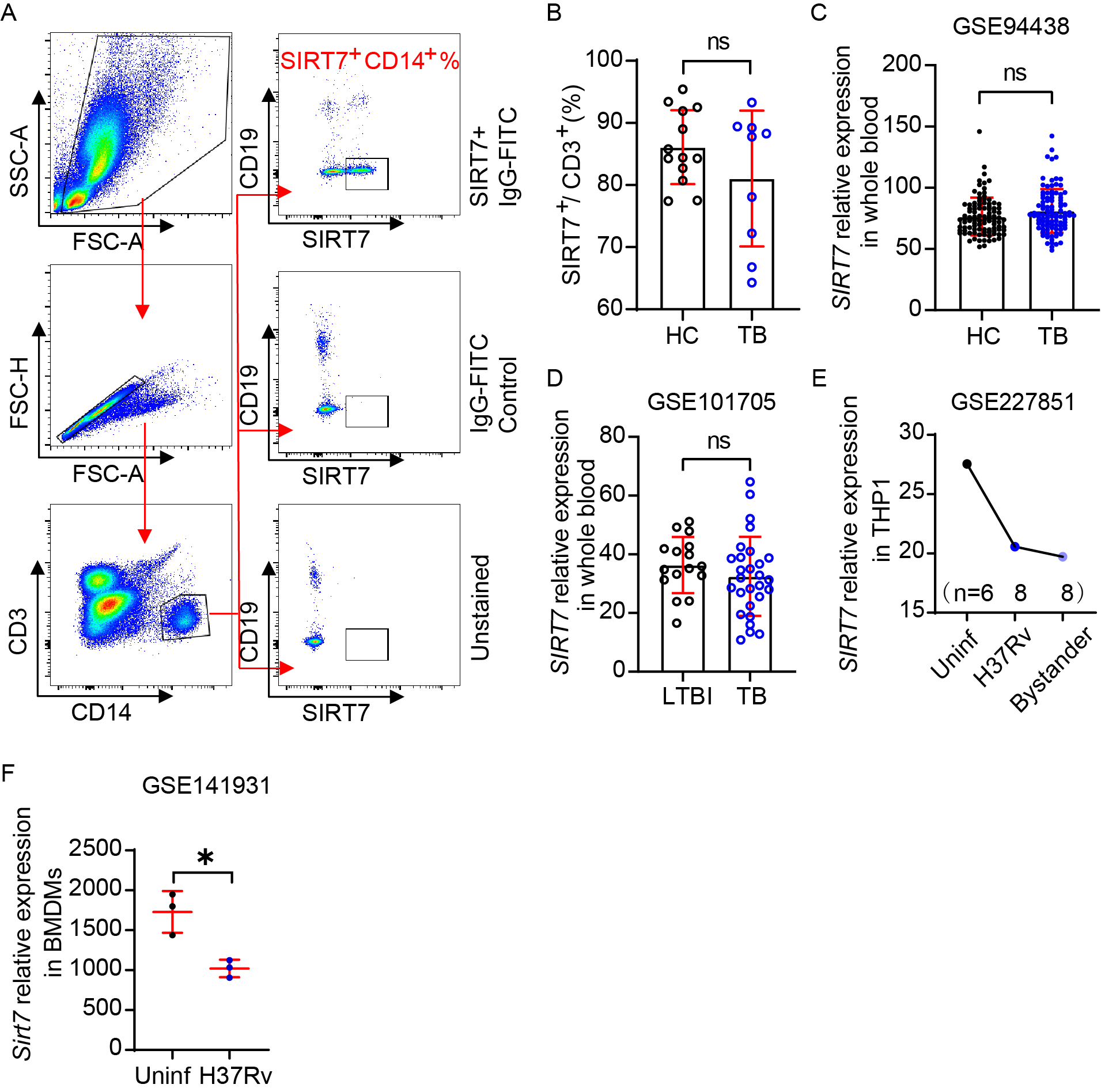


**FIG S1** Efficient identification of SIRT7-positive monocytes via indirect flow cytometry and SIRT7 downregulation post-*Mtb* infection. (A) Flow cytometry analysis of SIRT7 expression in monocytes (identified after excluding CD3^+^ T cells, CD19^+^ B cells, and duplicates) from peripheral blood using anti-CD3, anti-CD14, anti-CD19, and anti-SIRT7 antibodies followed by FITC-conjugated secondary IgG antibody. Control samples were either stained with the secondary antibody alone or left unstained. (B) Quantification of SIRT7 expression in T cells (CD3^+^) from HC subjects (n = 13) and TB patients (n = 10). (C) TPM-normalized expression values of the *SIRT7* in whole blood from HC (n=98) and TB (n=98) subjects, based on the GSE94438 dataset. (D) TPM-normalized expression values of *SIRT7* in whole blood from LTBI (n=16) and TB (n=28) subjects, based on the GSE94438 dataset. (E) Microarray data (GSE227851) visualization showing *SIRT7* expression levels in THP1-derived macrophages and bystander cells (Cells in the infected samples that have not phagocytized H37Rv) following H37Rv infection. (F) RNA-seq data (GSE141931) visualization indicating *Sirt7* expression levels in BMDMs post-infection. Data are presented as means ± SEM; ns, not significant; *P < 0.01, as determined by Student’s two-tailed unpaired t-test.


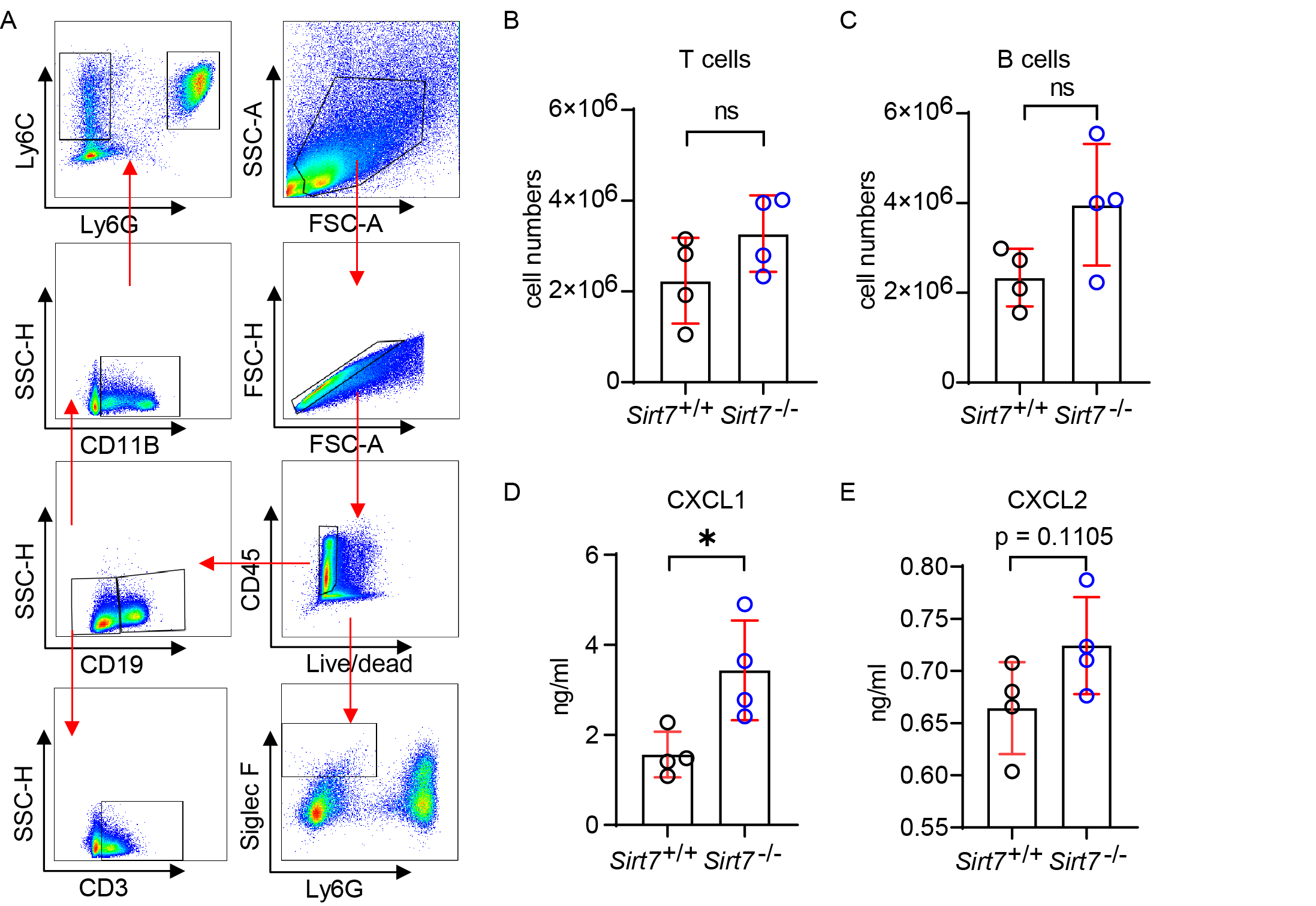


**FIG S2** SIRT7 deficiency significantly increases the absolute number of neutrophils in the lungs of the TB mouse model. (A) Gating strategies utilized to identify various immune cell populations: monocytes (CD45^+^CD19^-^CD11b^+^Ly6G^+^Ly6C^+^), alveolar macrophages (CD45^+^ Ly6G^+^siglecf^+^), T cells (CD45^+^CD19^-^CD3^+^), B cells (CD45^+^CD19^+^), and neutrophils (CD45^+^CD19^-^CD11b^+^Ly6G^+^Ly6C^+^) post-exclusion of duplicate and dead cells (FSC/SSC/Zombie Aqua^+^). (B and C) Absolute numbers of T cells and B cells in the lungs of H37Rv-infected *Sirt7*^−/−^ and *Sirt7*^+/+^ mice are depicted. (D and E) ELISA detected elevated levels of CXCL1 and CXCL2 in lung homogenate supernatants from *Sirt7*^−/−^ compared to *Sirt7*^+/+^ mice. Data are presented as means ± SEM; ns, not significant; *P < 0.01, as determined by Student’s two-tailed unpaired t-test. Each experiment was independently replicated three times.


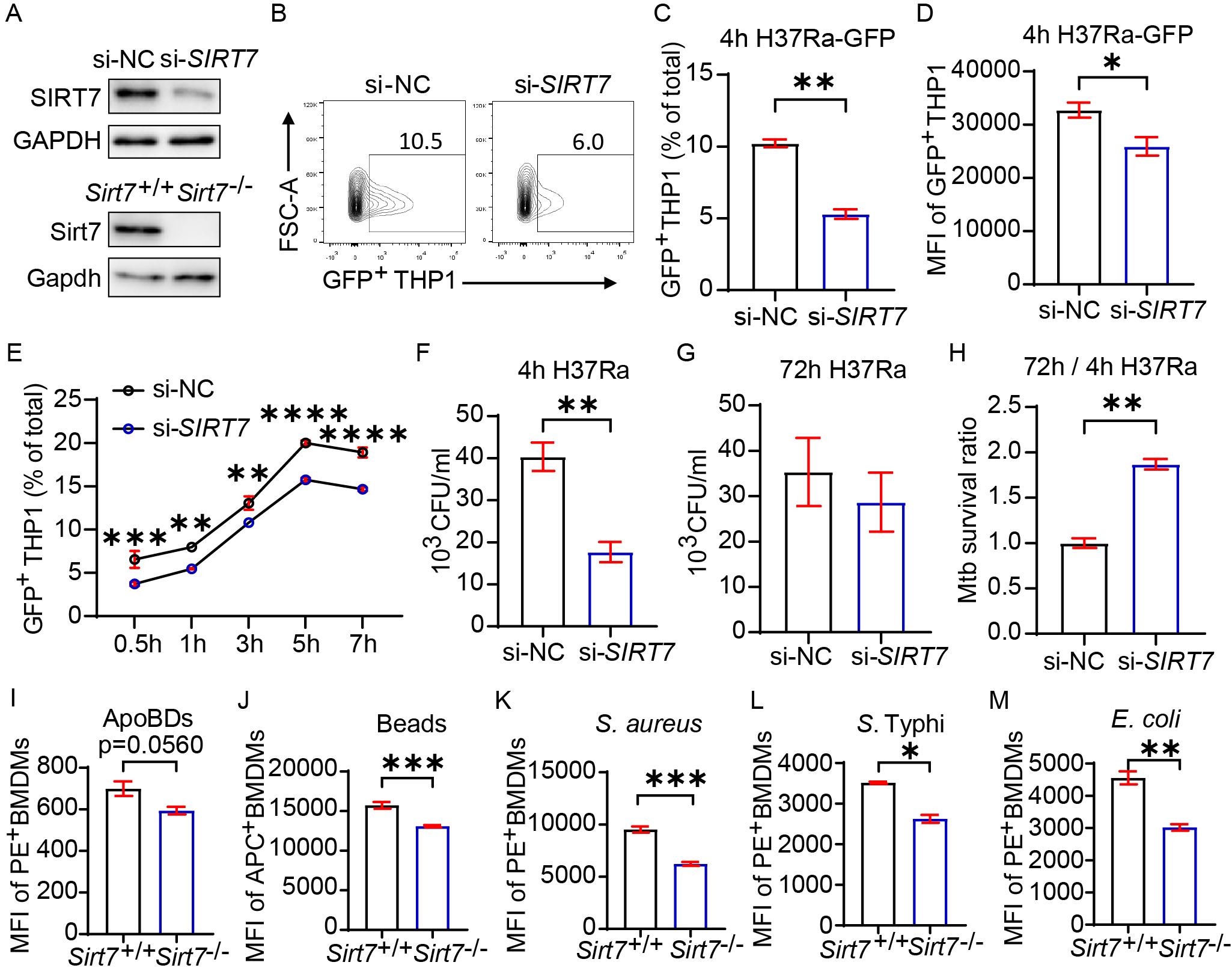


**FIG S3** SIRT7 deficiency broadly affects macrophage phagocytic activity beyond *Mtb*. (A) Immunoblot analysis demonstrating SIRT7 levels in THP1-derived macrophages transfected with *SIRT7* siRNA (upper) and those in *Sirt7*^+/+^ and *Sirt7*^−/−^ BMDMs (lower). (B through D) Flow cytometry analyses and quantification of GFP-H37Ra phagocytosis by *SIRT7*-knockdown in THP1-derived macrophages compared with wild-type (WT) controls. (E) Flow cytometry quantification of phagocytosis of GFP-H37Ra by *SIRT7* -knockdown THP1-derived macrophages at various time points (0.5, 1, 3, 5, and 7 h). (F and G) Intracellular CFU counts of H37Ra assessed 4 h or 72 h post-infection in *SIRT7*-knockdown THP1-derived macrophages and WT counterparts. (H) Survival ratio of H37Ra were evaluated in SIRT7-knockdown THP1-derived macrophages compared to WT controls. (I through M) Mean fluorescence intensity of *Sirt7*^−/−^ and *Sirt7*^+/+^ BMDMs post-phagocytosis of apoptotic bodies, beads, *Staphylococcus aureus, Escherichia coli*, or *Salmonella* Typhi. Data are presented as means ± SEM, *P < 0.05, **P < 0.01, ***P < 0.001, ****P < 0.0001, as analyzed by two-way ANOVA with Sidak's multiple comparisons test (E) or Student’s two-tailed unpaired t-test (C, D, F-M).


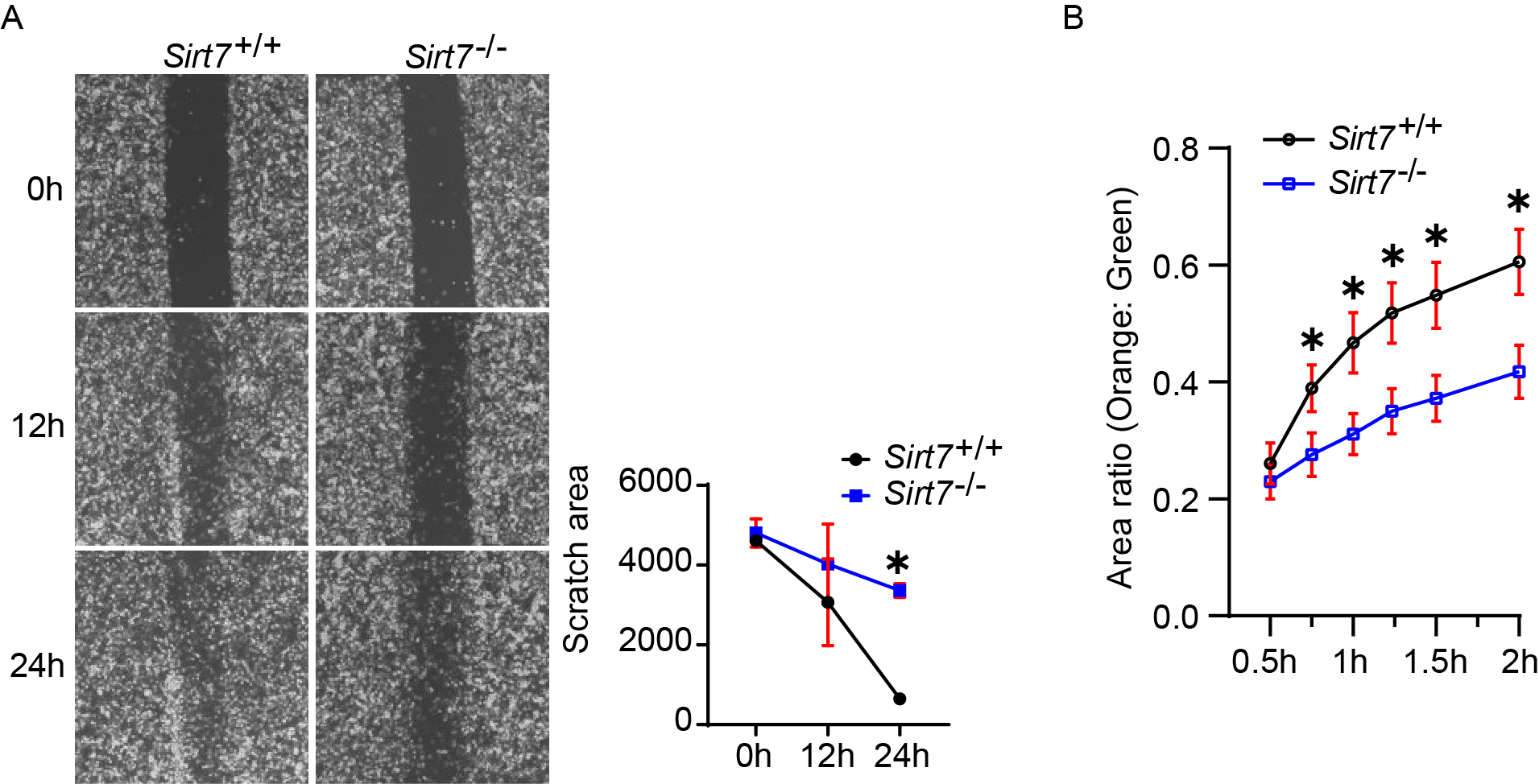


**FIG S4**  SIRT7 deficiency alters macrophage phagocytosis and migration dynamics. (A) Migration of *Sirt7*^−/−^ and *Sirt7*^+/+^ BMDMs, as assessed using a wound closure (scratch) assay. (B) Time-lapse microscopy was utilized to capture the phagocytosis process of pHrodo Red-labeled GFP-H37Ra (shifting from green to orange upon phagocytosis) by *Sirt7*^−/−^ and *Sirt7*^+/+^ BMDMs. Line graph showing the ratio of phagocytosed GFP-H37Ra (orange) to total GFP-H37Ra (green) at 0.5, 1, 1.5, and 2 h post-infection in *Sirt7*^−/−^ and *Sirt7*^+/+^ BMDMs. Data are presented as means ± SEM, *P < 0.05, as analyzed by two-way ANOVA with Sidak's multiple comparisons test.


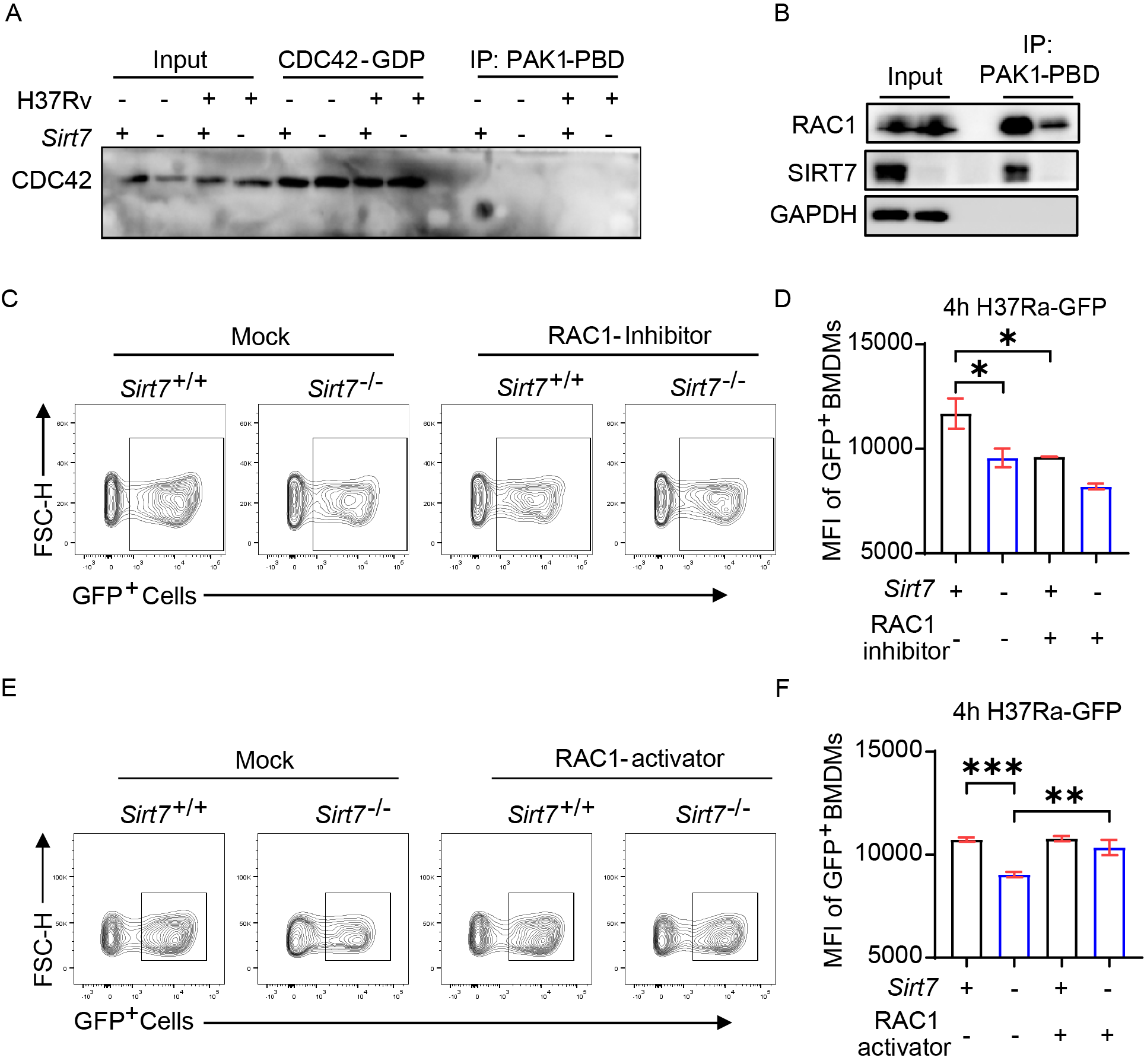


**FIG S5** SIRT7 interacts with PAK1-PBD. (A) CDC42 activation in H37Rv-infected *Sirt7*^−/−^ and *Sirt7*^+/+^ BMDMs at 0 min and 30 min post-infection. Immunoblotting did not detect Pak1-PBD pulldown of CDC42. (B) Immunoprecipitation (IP) assay of RAC1 activation in *Sirt7*^−/−^ and *Sirt7*^+/+^ BMDMs. PAK1-PBD interacted not only with GTP-bound RAC1 but also with Sirt7 in BMDMs. (C through F) Flow cytometry analysis of GFP-H37Ra phagocytosis in *Sirt7*^−/−^ and *Sirt7*^+/+^ BMDMs under conditions of RAC1 inhibitor or activator treatment.


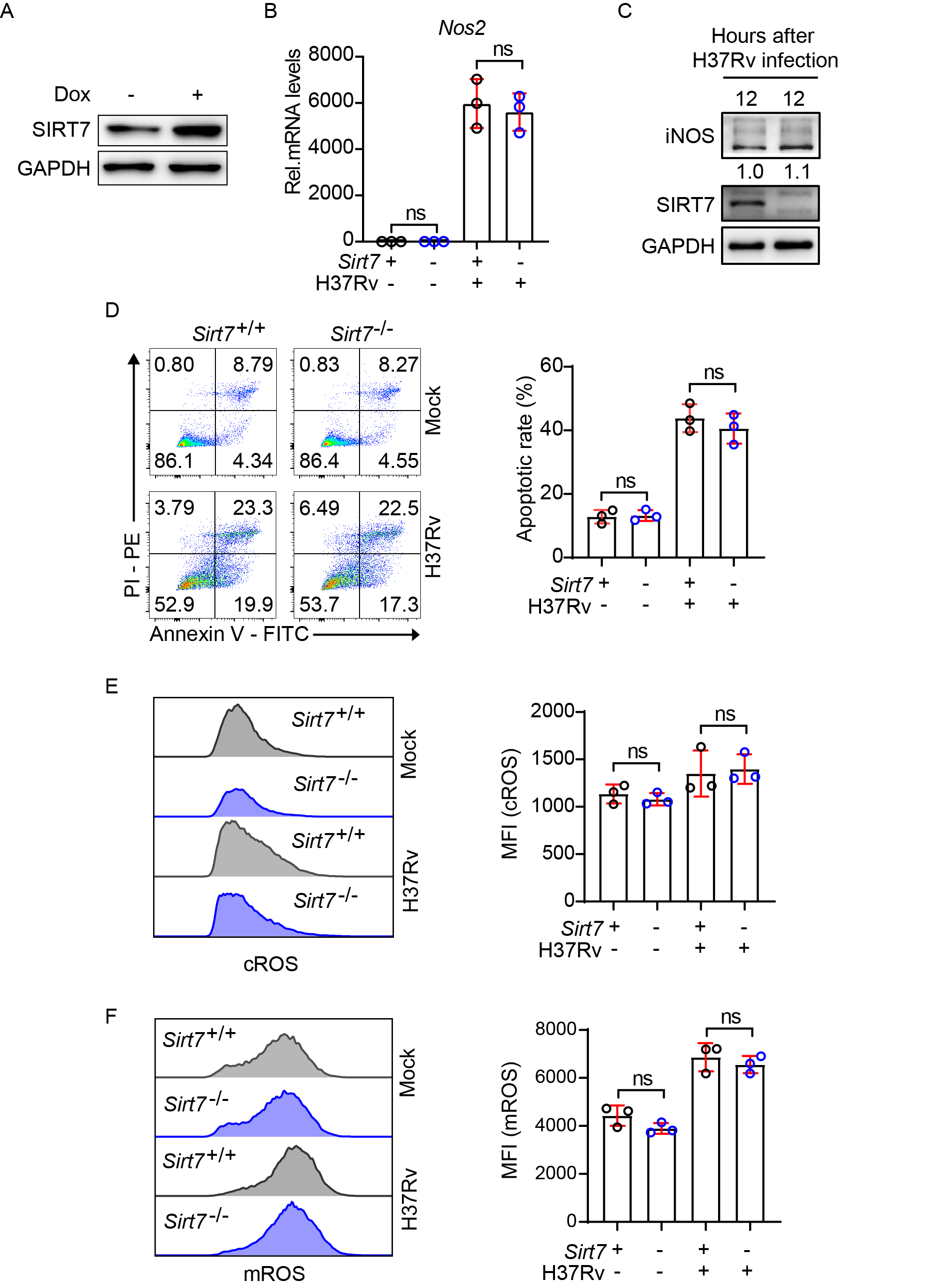


**FIG** **S6.** SIRT7 does not affect iNOS expression, apoptosis and ROS in BMDMs. (A) Immunoblot analysis of SIRT7 in *Sirt7*-transgenic mouse (*Sirt7*^TG^) BMDMs, with or without Dox induction. (B) Expression of the *Nos2* gene across various macrophages; (C) Expression of iNOS protein in *Sirt7*^+/+^ and *Sirt7*^−/−^ BMDMs infected with H37Rv. (D) Flow cytometric analysis of apoptosis in H37Rv-infected *Sirt7*^+/+^ and *Sirt7*^−/−^ BMDMs. (E and F) Flow cytometric analysis of cROS and mROS in H37Rv-infected *Sirt7*^+/+^ and *Sirt7*^−/−^ BMDMs. Data are represented as means ± SEM; ns, not significant, as determined by Student’s two-tailed unpaired t-test.


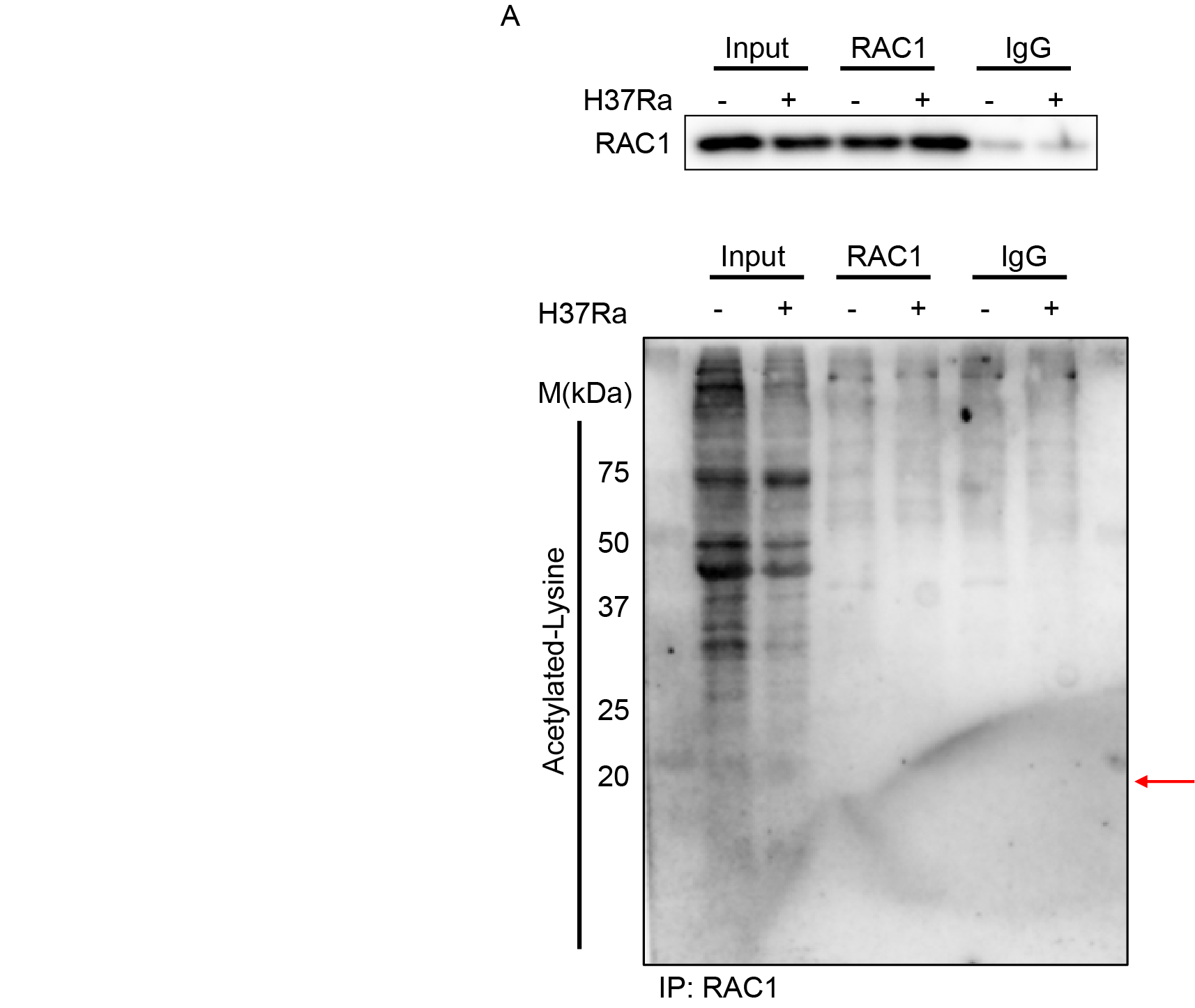
**FIG S7** No acetylation of RAC1 was detected in both *Mtb*-infected and uninfected BMDMs. After enriching RAC1 protein from BMDMs treated under various conditions using a RAC1 antibody, the acetylation of RAC1 was detected using an Acetylated-Lysine Antibody.
