# Supplementary material for: SIRT7 remodels the cytoskeleton via RAC1 to enhance host resistance to Mycobacterium tuberculosis
Source: mBio. 2024 Sep 17;15(10):e00756-24. doi: 10.1128/mbio.00756-24 (PMC11481912; doi:10.1128/mbio.00756-24)
Supplement: Supplemental Tables — Tables S1 to S3. [file mbio.00756-24-s0002.docx]

**Table S1 Differentially Expressed Genes in BMDMs following *Sirt7* Knockout under H37Rv Infection**

| **SYMBOL** | ***Sirt7*^-/-^ RV** | ***Sirt7*^+/+^ RV** | **log2FoldChange** | ***p*value** | ***p*adj** |
| --- | --- | --- | --- | --- | --- |
| Igkv17-127 | 0.894541 | 58.20023 | -6.03325 | 4.85E-08 | 8.12E-05 |
| Igkv8-30 | 0.530444 | 37.92342 | -6.02117 | 1.71E-06 | 0.000846 |
| Gm15446 | 0.631835 | 30.95425 | -5.70828 | 1.43E-07 | 0.000182 |
| Igkv6-15 | 0.897057 | 21.74226 | -4.60874 | 0.000306 | 0.046581 |
| Jchain | 38.70074 | 631.7131 | -4.02982 | 7.88E-05 | 0.019534 |
| Igkc | 203.9929 | 2483.126 | -3.60588 | 5.27E-06 | 0.002203 |
| Igha | 92.39088 | 1084.478 | -3.55331 | 0.000176 | 0.032764 |
| Ighg2b | 58.0849 | 590.9469 | -3.34325 | 1.57E-15 | 7.02E-12 |
| Iglc2 | 2.861125 | 23.25429 | -2.99224 | 0.000168 | 0.032654 |
| Ighm | 23.77968 | 154.0582 | -2.69601 | 1.50E-07 | 0.000182 |
| G530011O06Rik | 37.38946 | 212.3134 | -2.507 | 1.27E-21 | 8.49E-18 |
| Dscc1 | 6.494709 | 28.79122 | -2.1421 | 1.16E-05 | 0.004686 |
| 3830403N18Rik | 20.60798 | 65.99285 | -1.68247 | 4.52E-06 | 0.001951 |
| Syt4 | 0.62289 | 1.872925 | -1.63152 | 0.242086 |  |
| Dct | 0.315918 | 1.108806 | -1.60819 | 0.250589 | 0.960605 |
| Cbr2 | 69.64581 | 202.8538 | -1.54145 | 1.45E-08 | 3.23E-05 |
| Hpgd | 21.64566 | 61.26804 | -1.50928 | 3.80E-07 | 0.0003 |
| Rarres1 | 46.39569 | 118.8795 | -1.3576 | 0.000199 | 0.03496 |
| Spc24 | 23.38328 | 56.21618 | -1.27385 | 0.000141 | 0.029066 |
| 2610035D17Rik | 11.1181 | 26.24737 | -1.25608 | 0.000603 | 0.06726 |
| Agt | 23.69366 | 56.35427 | -1.24683 | 6.97E-05 | 0.017601 |
| C130026I21Rik | 49.37602 | 115.794 | -1.22629 | 3.63E-08 | 6.94E-05 |
| Calhm5 | 37.09345 | 85.53594 | -1.2042 | 0.000221 | 0.0365 |
| Lmod1 | 24.40157 | 56.24287 | -1.19219 | 0.000173 | 0.032764 |
| Rspo2 | 52.79362 | 120.9828 | -1.1883 | 1.81E-07 | 0.000186 |
| Sytl2 | 20.56291 | 46.61426 | -1.17005 | 3.41E-05 | 0.010864 |
| Pimreg | 59.87673 | 130.273 | -1.12171 | 1.17E-06 | 0.000672 |
| Avpr1a | 46.12999 | 98.30707 | -1.09564 | 5.40E-05 | 0.015317 |
| Dpep1 | 89.48102 | 175.3332 | -0.97173 | 1.94E-07 | 0.000186 |
| Sbsn | 115.0739 | 221.4242 | -0.94497 | 0.00011 | 0.025469 |
| Gas1 | 43.31918 | 82.78636 | -0.93424 | 1.51E-05 | 0.005947 |
| Mxra7 | 29.38876 | 55.88937 | -0.92493 | 0.000222 | 0.0365 |
| Cxcl12 | 2297.308 | 4317.032 | -0.90987 | 1.92E-07 | 0.000186 |
| Ercc6l | 61.98554 | 114.2871 | -0.88413 | 0.000318 | 0.046771 |
| Ndc80 | 54.40682 | 100.2292 | -0.88146 | 4.95E-05 | 0.014418 |
| Ttk | 40.51832 | 74.58735 | -0.87906 | 0.000136 | 0.028544 |
| Tk1 | 139.1808 | 254.9906 | -0.87381 | 0.000124 | 0.027688 |
| Islr | 92.60071 | 169.2237 | -0.86643 | 1.79E-05 | 0.0068 |
| Fbxo5 | 52.24752 | 94.94943 | -0.85959 | 4.82E-05 | 0.014339 |
| Nkd2 | 128.9296 | 234.038 | -0.85803 | 8.74E-05 | 0.020893 |
| Cdkn1c | 204.0828 | 363.8408 | -0.83662 | 3.27E-05 | 0.01067 |
| Ccna2 | 204.1887 | 364.3264 | -0.83633 | 1.28E-06 | 0.000672 |
| Tmem47 | 211.159 | 371.6524 | -0.81354 | 1.83E-05 | 0.0068 |
| Acta2 | 5154.518 | 8874.443 | -0.78386 | 0.000259 | 0.040754 |
| Ckap2 | 85.82028 | 145.8262 | -0.76445 | 0.000147 | 0.029816 |
| Anln | 159.6924 | 271.7244 | -0.76279 | 5.28E-07 | 0.000353 |
| Cdc20 | 125.3994 | 209.3988 | -0.74291 | 0.000135 | 0.028544 |
| Bicc1 | 218.5558 | 365.4275 | -0.74035 | 4.27E-06 | 0.001906 |
| Parva | 490.1073 | 817.3975 | -0.73835 | 0.000198 | 0.03496 |
| Epdr1 | 199.6196 | 332.0388 | -0.73488 | 0.000118 | 0.026675 |
| Ckap2l | 118.3711 | 197.396 | -0.73443 | 4.82E-05 | 0.014339 |
| Kif11 | 81.42895 | 133.8571 | -0.71366 | 3.23E-05 | 0.01067 |
| Mark1 | 170.461 | 278.1093 | -0.70337 | 0.000224 | 0.0365 |
| Racgap1 | 156.5928 | 253.6388 | -0.69819 | 1.30E-06 | 0.000672 |
| Gypc | 420.5726 | 680.9897 | -0.69504 | 0.000331 | 0.048102 |
| Sdc2 | 596.1575 | 963.2094 | -0.6926 | 0.000344 | 0.049565 |
| Sirt7 | 391.9087 | 631.4539 | -0.68737 | 4.72E-05 | 0.014339 |
| Colec12 | 705.3057 | 1134.437 | -0.68451 | 2.69E-10 | 7.20E-07 |
| Vgll3 | 832.6095 | 1328.885 | -0.67419 | 0.000136 | 0.028544 |
| Antxr1 | 836.8784 | 1322.505 | -0.65972 | 2.88E-05 | 0.009881 |
| Trip6 | 248.1336 | 384.924 | -0.63247 | 0.000166 | 0.032654 |
| Smco4 | 102.1239 | 157.9639 | -0.62847 | 0.000254 | 0.040461 |
| Ogn | 45.70586 | 70.31439 | -0.61696 | 0.008558 | 0.319985 |
| Cdkn2c | 129.164 | 197.8034 | -0.61612 | 0.000291 | 0.044705 |
| Mcm7 | 263.2755 | 403.1782 | -0.61513 | 3.19E-06 | 0.00147 |
| Pdgfra | 463.971 | 705.5557 | -0.60419 | 8.03E-05 | 0.019534 |
| Rhoj | 183.811 | 278.0727 | -0.59786 | 0.00031 | 0.046682 |
| Col16a1 | 247.0369 | 373.4842 | -0.59441 | 0.000133 | 0.028544 |
| Fbxo32 | 252.5628 | 380.9924 | -0.59089 | 5.61E-05 | 0.015317 |
| Tmem178 | 135.8557 | 193.5265 | -0.51028 | 0.016604 | 0.468908 |
| Il12a | 643.7726 | 428.2863 | 0.588514 | 0.000201 | 0.03496 |
| Bst1 | 4282.356 | 2821.022 | 0.602246 | 2.33E-07 | 0.000208 |
| Fhip2b | 260.6193 | 169.7886 | 0.616703 | 0.00015 | 0.030025 |
| Vill | 235.3715 | 141.664 | 0.732361 | 0.000192 | 0.034763 |
| Arfgef3 | 122.82 | 70.4616 | 0.79696 | 0.000316 | 0.046771 |
| Mcoln3 | 464.7592 | 266.3622 | 0.802837 | 4.96E-07 | 0.00035 |
| Tyrp1 | 0.315918 | 0 | 0.836447 | 0.604784 | 0.960605 |
| Tmem267 | 280.1593 | 153.6159 | 0.867485 | 0.000174 | 0.032764 |
| Adam33 | 114.7991 | 62.38281 | 0.879273 | 0.000273 | 0.042478 |
| Rgs18 | 73.44553 | 38.58067 | 0.924312 | 5.90E-05 | 0.015483 |
| Ccl22 | 561.7084 | 290.6421 | 0.94933 | 4.51E-07 | 0.000335 |
| Cd59a | 199.9365 | 98.81093 | 1.014398 | 3.81E-07 | 0.0003 |
| Zfp268 | 173.0593 | 71.71021 | 1.271945 | 2.29E-06 | 0.001095 |
| Xlr3b | 60.61336 | 23.42847 | 1.381037 | 1.24E-06 | 0.000672 |
| Wdfy1 | 1085.034 | 342.6726 | 1.663878 | 6.84E-25 | 9.15E-21 |
| Rab4a | 87.07164 | 25.90382 | 1.751619 | 2.65E-11 | 8.88E-08 |
| Gm16867 | 49.58793 | 0.34526 | 6.98791 | 5.60E-08 | 8.33E-05 |

**Table S2 Differentially Expressed Genes in BMDMs following *Sirt7* Knockout**

| **SYMBOL** | **Sirt7-/-** | **Sirt7+/+** | **log2FoldChange** | **p-value** | **padj** |
| --- | --- | --- | --- | --- | --- |
| Igkv8-30 | 0.424735 | 31.31212 | -6.28288 | 0.000182 | 0.065364 |
| Gm15446 | 0.849469 | 39.16643 | -5.60079 | 5.48E-07 | 0.000463 |
| Igkv6-15 | 0.379278 | 16.56962 | -5.38452 | 0.00328 | 0.594164 |
| Igkv17-127 | 1.51711 | 41.5759 | -4.73703 | 2.96E-05 | 0.016676 |
| Il6 | 1.228747 | 20.43184 | -4.0675 | 6.41E-06 | 0.004642 |
| Saa3 | 72.87675 | 765.2003 | -3.39309 | 1.65E-20 | 2.50E-16 |
| Cxcl3 | 8.889592 | 68.71319 | -2.95503 | 3.72E-14 | 1.37E-10 |
| Lcn2 | 1.987302 | 15.00208 | -2.85292 | 5.35E-05 | 0.024679 |
| Acod1 | 29.703 | 145.5878 | -2.29767 | 3.28E-08 | 4.16E-05 |
| Ifi205 | 3.216049 | 15.34646 | -2.29713 | 5.04E-05 | 0.023982 |
| Iglc2 | 5.809914 | 24.18613 | -2.07123 | 0.014885 | 1 |
| Igha | 150.7949 | 567.9742 | -1.91281 | 0.090648 | 1 |
| Il12a | 0 | 0.49991 | -1.86359 | 0.294085 | 1 |
| Jchain | 112.6256 | 408.4683 | -1.85816 | 0.125723 | 1 |
| H2-M2 | 20.19122 | 67.32954 | -1.743 | 8.71E-08 | 8.83E-05 |
| Igkc | 424.1917 | 1328.585 | -1.64699 | 0.081413 | 1 |
| Il1a | 27.89752 | 83.05367 | -1.57919 | 0.000131 | 0.049742 |
| 3830403N18Rik | 21.23814 | 62.5915 | -1.55366 | 0.000264 | 0.089341 |
| C130026I21Rik | 14.80604 | 40.77417 | -1.46168 | 1.47E-05 | 0.008969 |
| Il1b | 136.3028 | 373.6092 | -1.45532 | 3.60E-12 | 6.84E-09 |
| Marco | 25.48549 | 67.52478 | -1.41645 | 1.26E-05 | 0.008129 |
| Ighg2b | 161.4417 | 416.7495 | -1.36806 | 0.005312 | 0.810416 |
| Rsad2 | 46.54179 | 117.7548 | -1.34373 | 8.33E-05 | 0.035199 |
| 2610035D17Rik | 75.0615 | 187.9652 | -1.32265 | 5.28E-06 | 0.004015 |
| Cxcl2 | 48.72655 | 117.2848 | -1.26385 | 4.94E-08 | 5.79E-05 |
| Tmem178 | 29.14189 | 68.84414 | -1.23762 | 3.54E-05 | 0.017537 |
| Sirt7 | 364.3553 | 852.6899 | -1.22754 | 7.47E-10 | 1.14E-06 |
| Ifit1 | 44.91815 | 102.5451 | -1.19598 | 5.88E-05 | 0.026296 |
| Slamf8 | 189.8222 | 410.7667 | -1.11482 | 3.39E-05 | 0.017537 |
| Dcstamp | 85.62019 | 183.9726 | -1.09968 | 3.55E-05 | 0.017537 |
| Siglecf | 183.2239 | 380.9058 | -1.05608 | 6.67E-10 | 1.13E-06 |
| F10 | 135.1209 | 273.5657 | -1.01894 | 3.84E-06 | 0.003073 |
| Procr | 104.4165 | 208.0692 | -0.99567 | 1.15E-08 | 1.60E-05 |
| Itgax | 1495.819 | 2954.145 | -0.98189 | 5.89E-08 | 6.41E-05 |
| Ighm | 188.6105 | 362.1158 | -0.94129 | 0.105537 | 1 |
| Mmp12 | 11430.01 | 21182.4 | -0.89002 | 1.15E-13 | 2.92E-10 |
| Egr2 | 446.6387 | 736.0376 | -0.71855 | 0.000103 | 0.040026 |
| Dscc1 | 42.52173 | 69.64197 | -0.70545 | 0.110564 | 1 |
| Cd300ld | 1503.15 | 2443.905 | -0.70058 | 1.28E-05 | 0.008129 |
| Rarres1 | 74.62114 | 117.1229 | -0.64719 | 0.125257 | 1 |
| Ccr1 | 515.7511 | 807.6184 | -0.64678 | 9.33E-05 | 0.03737 |
| Sbsn | 146.4666 | 99.87433 | 0.548604 | 0.058569 | 1 |
| Epdr1 | 551.0566 | 372.1397 | 0.565543 | 0.009365 | 1 |
| Cd59a | 453.5438 | 296.6275 | 0.615012 | 0.003891 | 0.665265 |
| Vill | 228.2343 | 147.9646 | 0.621058 | 0.006054 | 0.87811 |
| Arfgef3 | 202.7929 | 131.657 | 0.622857 | 0.008421 | 1 |
| Adam33 | 81.16118 | 53.00456 | 0.628771 | 0.025614 | 1 |
| Mcoln3 | 1719.984 | 1106.686 | 0.637122 | 0.000328 | 0.10395 |
| Hpgd | 1308.827 | 826.8746 | 0.662845 | 0.011425 | 1 |
| Ogn | 588.3523 | 325.7646 | 0.851364 | 8.80E-05 | 0.0362 |
| Wif1 | 174.2888 | 74.87296 | 1.210838 | 7.50E-05 | 0.032591 |
| Xlr3b | 63.80533 | 27.14347 | 1.232124 | 3.57E-05 | 0.017537 |
| Xlr | 98.2557 | 31.80927 | 1.616017 | 9.71E-08 | 9.24E-05 |
| Wdfy1 | 816.061 | 260.2881 | 1.650892 | 5.47E-18 | 4.16E-14 |
| Rab4a | 194.5425 | 48.48982 | 2.000014 | 1.30E-15 | 6.60E-12 |
| Pmel | 73.53017 | 6.404931 | 3.517404 | 3.71E-07 | 0.000332 |
| Gm16867 | 9.693604 | 0 | 6.016392 | 9.14E-06 | 0.006324 |
| Syt4 | 21.48104 | 0.249955 | 6.206614 | 2.55E-05 | 0.01494 |
| Dct | 93.91884 | 0 | 9.296352 | 1.35E-12 | 2.94E-09 |
| Tyrp1 | 125.3662 | 0 | 9.712791 | 4.51E-14 | 1.37E-10 |

**Table S3. Characteristics of patients with active TB and healthy controls**

|  | Healthy | Active TB | *P*-value |
| --- | --- | --- | --- |
| Sample size (no.) | 17 | 15 | – |
| Age (years) (mean ± SD) | 40.2 ± 12.89 | 36.4 ± 12.75 | 0.3972 |
| Sex (M/F) | 11/6 | 9/6 | 1 |

F, Female; M, male. The level of significance was evaluated by unpaired student t-test or Chi-square test. P-value < 0.05 was considered statistically significant.
